# Development of a Charge-Implicit ReaxFF for C/H/O Systems

**DOI:** 10.1021/acs.jpclett.1c03867

**Published:** 2022-01-12

**Authors:** Michał Kański, Sviatoslav Hrabar, Adri C. T. van Duin, Zbigniew Postawa

**Affiliations:** † Smoluchowski Institute of Physics, Jagiellonian University, Łojasiewicza 11, 30-348 Kraków, Poland; ‡ Department of Mechanical Engineering, Pennsylvania State University, University Park, Pennsylvania 16802, United States

## Abstract

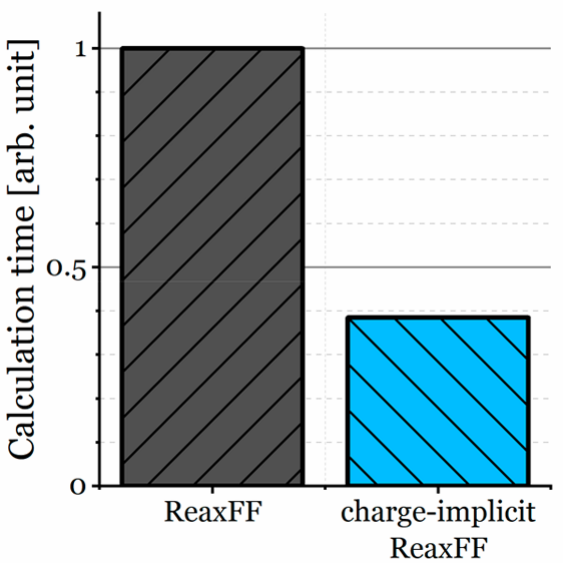

Modeling chemical reactions in condensed phases is difficult. Interaction potentials (or force fields) like ReaxFF can perform this modeling with a high overall accuracy, but the disadvantage of ReaxFF is a low simulation speed arising from costly algorithms, in particular charge equilibration. Therefore, we reparametrized ReaxFF to incorporate Coulomb forces into other terms of the force field. Because of this change, our charge-implicit ReaxFF-CHO is >2 times faster than the original parametrization. Despite the lack of explicit electrostatic interactions, our potential can correctly model the reactions and densities of systems containing carbon, hydrogen, and oxygen atoms. We have used the new potential to simulate bombardment of trehalose by water clusters. It has been observed experimentally that these water projectiles can increase the sensitivity of secondary ion mass spectrometry by more than an order of magnitude, but no explanation for this phenomenon was given. Our simulations show that the increase in the intensity of the recorded signal coincides with the emission of trehalose–water complexes.

At the heart of every molecular dynamics (MD) computer simulation is an interatomic potential (force field) used to describe the interactions between atoms in a modeled system. ReaxFF is one of the most widely utilized potentials for modeling organic systems undergoing chemical reactions.^1^ It allows for an accurate description of forces in complex systems. However, compared to most other force fields, ReaxFF has the disadvantage of a relatively high computational cost. To circumvent this issue and increase the applicability of the potential to high-energy sputtering simulations, we had previously developed the charge-implicit ReaxFF for hydrocarbon systems (ci-ReaxFF).^2^ Because of the inclusion of the effect of the electrostatic interactions in other terms of the ReaxFF formalism, we achieved 2–5 times faster simulations without a significant loss of computational accuracy. However, there are already other potentials, such as AIREBO,^3^ that can describe chemical reactions in hydrocarbon systems without explicit Coulomb forces. A much greater challenge would be an extension of the ci-ReaxFF potential to systems containing oxygen, where the effect of the electrostatic forces is much more pronounced, especially for nonbonding interactions. To the best of our knowledge, there are no reactive force fields that can properly describe both chemical reactions and densities of materials containing oxygen without explicit Coulomb interactions. Therefore, in this work, we present the first charge-implicit reactive potential, which can model chemical reactions for systems composed of carbon (C), hydrogen (H), and oxygen (O). Additionally, it can accurately predict densities for a wide range of molecular systems and incorporates proper close-range repulsive barriers, which allows modeling of high-energy collisions. We have used the new potential to gain insight into the processes involved in sputtering of organic systems by massive water clusters. It has been reported that such projectiles may increase the ionization probability by more than an order of magnitude, which significantly improves the sensitivity of secondary ion mass spectrometry (SIMS).^4^ However, this boosting effect is present only in a very narrow window of kinetic energy per cluster molecule and the mechanism standing behind it remains unknown. The simulations with the ci-ReaxFF-CHO potential will shed light on this phenomenon.

The parameters of the interactions among oxygen, hydrogen, and carbon atoms were fitted using a procedure similar to that in the original ci-ReaxFF publication.^2^ Briefly, the bonding parameters of the potential were optimized by a successive single-parameter search algorithm.^1^ Next, the densities and cohesive energies were computed, and the nonbonding parameters were adjusted so that the difference between the computed and reference values was <10% in general. After that, the bonding parameters were refitted, and the simulations of densities and cohesive energies were performed anew. If the deviation from the reference value was still <10%, the procedure had reached completion. Otherwise, the nonbonding parameters were further adjusted. All simulations have been performed in LAMMPS,^5,6^ and VMD was used for visualizations.^7^

The reference data (training set) from the ReaxFF parametrization created by Chenoweth et al. (ReaxFF-2008)^8^ were used in the fitting procedure. It was augmented by densities of systems listed in Table 1 as well as the heat of vaporization of water and cohesive energy of amorphous ice. The reference value for the cohesive energy of amorphous ice was estimated to be 12.8 kcal/mol on the basis of the lattice enthalpy of cubic ice^9^ and the enthalpy of transformation from amorphous ice to cubic ice.^10^

**Table 1:** Comparison of Densities (in grams per cubic centimeter) and Cohesive Energies (in kilocalories per mole) of Selected Systems Predicted by ci-ReaxFF-CHO and Reax-lg^11^ to Experimental Values^a^

system	reference value	ci-Reax-CHO	Reax-lg
Density (g/cm^3^)			
(+)-camphor	0.99	0.98	1.05
1,5-pentanediol	0.99 (293 K)	1.09	1.09
1-octyne	0.75 (293 K)	0.73	0.78
2,6-xylenol	0.96 (293 K)	1.02	1.09
3-heptanol	0.82 (293 K)	0.85	0.90
acetophenone	1.03 (293 K)	1.01	1.11
benzene	0.88	0.79	0.89
benzyl acetate	1.06 (293 K)	1.03	1.17
butanoic anhydride	0.97 (293 K)	1.00	1.13
butyl methyl ether	0.74	0.77	0.85
cyclohexane	0.77	0.80	0.83
decane	0.73	0.77	0.80
dibutyl phthalate	1.05 (293 K)	0.99	1.17
diphenyl ether	1.07 (303 K)	1.00	1.16
ethane	0.54	0.51	0.53
ethyl acetate	0.90	1.08	1.07
indan	0.96 (293 K)	0.94	1.01
*m*-xylene	0.86	0.86	0.92
pentane	0.63 (293 K)	0.68	0.70
phenol	1.05 (318 K)	1.03	1.14
water	1.00	1.04	1.22
amorphous ice	0.94 (77 K)	1.00	1.36
**polyethylene**	0.92–0.97	0.85	0.89
**poly(methyl) methacrylate**	1.18	1.10	1.14
**polystyrene**	1.04–1.06	0.95	1.05
**trehalose**	1.58	1.42	1.54
**ethanol**	0.79	0.82	0.90
**water/ethanol(4:1)**(13)	0.81	0.86	0.74
**water/ethanol(1:1)**(13)	0.85	0.97	0.77
**water/ethanol(1:4)**(13)	0.94	1.13	gas
Cohesive Energy (kcal/mol)			
water	10.5	10.4	9.6
amorphous ice^9,10^	12.8 (77 K)	13.0	12.6

a The bold entries are molecules that were not part of the training set. The reference values were taken from ref (12) and were obtained at 298 K unless stated otherwise.

Table 1 shows the calculated densities and cohesive energies predicted by ci-ReaxFF-CHO compared with reference values and those from ReaxFF-lg, which predicts densities of materials with a reasonable accuracy.^11^ Inclusion of the electrostatic interactions in other terms did not stop the new potential from maintaining a good agreement with experimental values. The average deviation from the reference density values is 5%. This good overall agreement is mainly related to the hydrogen bond term in ReaxFF, which essentially replaces the short-range Coulomb terms. ci-ReaxFF-CHO correctly predicts an increase in the cohesive energy between liquid water and amorphous ice without a major change in density.

ci-ReaxFF-CHO maintains a good degree of accuracy for bond energies, as well as reaction, valence, and dihedral barriers. Figure 1 shows the bond dissociation curve for the abstraction of hydrogen from a water molecule (a), the hydrogen transfer barrier from hydrogen to the methyl group (b), the O–C–O valence barrier (c), and the O–C–C–O dihedral barrier (d). The first case is the most interesting as it proves that it is possible to maintain a good agreement with DFT data without explicit electrostatic interactions even for one of the most polar molecules. An average deviation from the training set values was 17%, compared to 16% achieved by ReaxFF-2008. Detailed comparison with DFT data is available in the Supporting Information.

**Figure 1:**
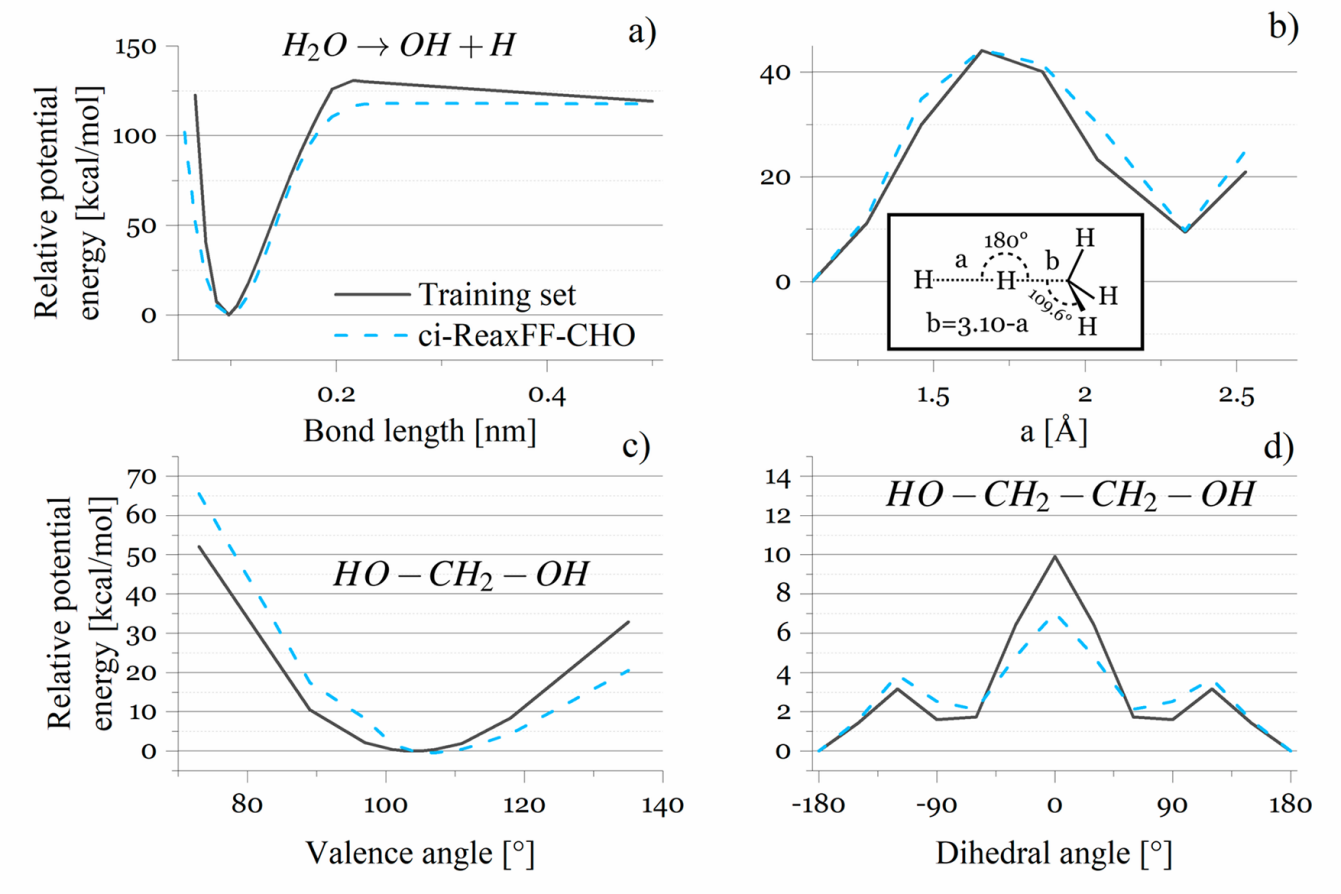
Comparison of (a) the O–H bond dissociation curve in water, (b) the hydrogen transfer barrier between hydrogen and the methyl group, (c) the O–C–O valence barrier in methanediol, and (d) the O–C–C–O dihedral barrier in ethylene glycol predicted by ci-ReaxFF-CHO (blue dashed lines) with reference values (black, solid lines).^8^

The last part of the fitting procedure was creation of tabularized close-range corrections to ci-ReaxFF-CHO to extend its agreement with the Ziegler–Biersack–Littmark (ZBL) potential, which is widely used for modeling close-range repulsive barriers.^14^ The procedure has been described extensively in ref (2). The values of inner and outer cutoffs for the transition region and a comparison between ci-ReaxFF-CHO and ZBL are given in the Supporting Information.

The new potential was subjected to a series of tests. First, the densities were modeled for molecules not included in the training set. Bold entries in Table 1 show the densities predicted by the new potential in comparison with reference values. The average deviation for test systems is larger than that obtained for the systems being a part of the training set and is equal to 10%. The densities of water/ethanol mixtures are overestimated; however, the potential correctly predicts an increase in the density with an increase in the ethanol fraction.^13^

To assess the applicability of the new potential for modeling of more energetic processes, we modeled oxidation of *o*-xylene in 2500 K. Similar simulations were performed with ReaxFF-2008,^8^ whose training set had been used during the fitting procedure. The details of the modeling protocol can be found in the Supporting Information. The oxidation was initiated by abstraction of a methyl hydrogen by an oxygen molecule, as in the case of simulations performed with ReaxFF-2008. The main products of oxidation were water, carbon monoxide, and carbon dioxide, the same as for ReaxFF-2008.

Finally, we tested the new potential by performing two simulations of amorphous ice bombardment by a 20 keV C_60_ projectile with a 40° incidence angle. The first one utilized ci-ReaxFF-CHO, and the second the second-generation ReaxFF water potential (Reax-H_2_O).^15^ This potential had been chosen because it was created specifically for modeling water. For the current simulations, this force field was augmented with a tabularized close-range repulsive potential, prepared like that for ci-ReaxFF-CHO. The simulation settings are available in the Supporting Information.

Figure 2 shows cross sections of both samples after the impact. The sizes of the produced craters are comparable; in both cases, a considerable number of water molecules were displaced creating a crater corona. The volumes of the ejected material (sputtering yield) were 72 and 67 nm^3^ for ci-ReaxFF-CHO and ReaxFF-H_2_O, respectively. Both values are close to the experimental result, which is 59 ± 6 nm^3^.^16^

**Figure 2:**
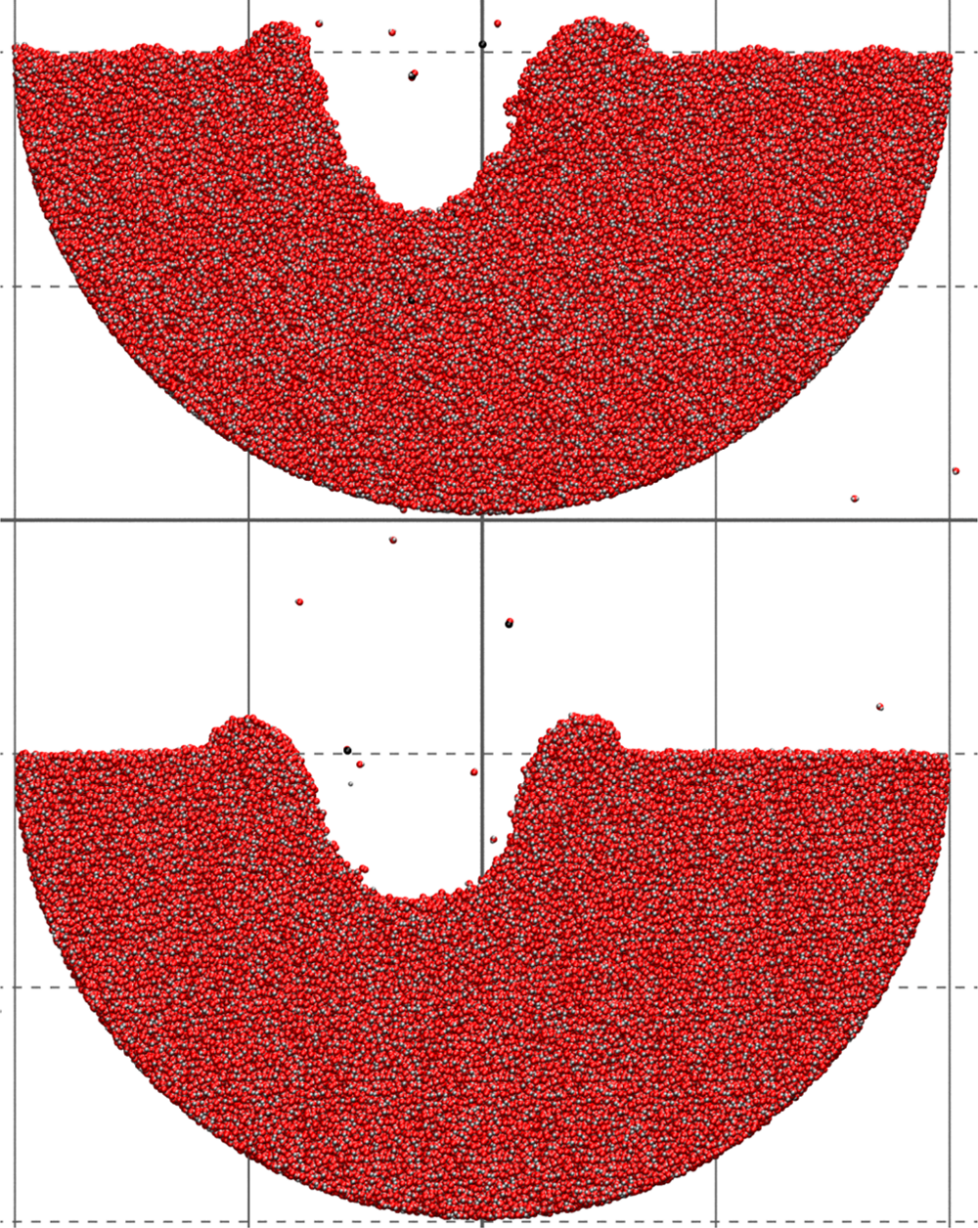
Cross sections (with a thickness of 3 nm) through craters after an impact of a 20 keV C_60_ projectile at a 40° incidence angle on amorphous ice modeled by ci-ReaxFF-CHO (top) and ReaxFF-H_2_O (bottom). The diameter of the samples is 40 nm.

The last important issue to discuss is the computational efficiency of the new potential compared to that of standard ReaxFF parametrizations. Table 2 shows the time of calculation and the acceleration for two simulations performed during the tests: density simulation of 1,5-pentanediol and impact of fullerene on frozen water. Additionally, the density simulation was again performed with a larger system. The time of calculation decreased 2.4 times for small systems and ∼2.7 times for larger simulations.

**Table 2:** Times of Calculation of the Chosen Simulations with ReaxFF-CHO and ReaxFF

		calculation time [μs (time step)^−1^ atom^–1^]		
system	no. of atoms	ci-ReaxFF-CHO	ReaxFF	acceleration (*x*-fold)
1,5-pentanediol	9728	4.60	11.1	2.4
	622592	2.09	5.85	2.8
water with C_60_	1672417/1743400	2.03	5.44	2.7

The primary purpose of our parametrization is its utilization for simulations of sputtering, which is the main phenomenon, for example, in SIMS.^17^ One of the long-standing conundrums of modern SIMS is an inexplicable increase in the intensity of the signal of positive molecular ions observed in experiments with massive water cluster projectiles for a narrow range of their kinetic energies. Figure 4 shows the phenomenon (the black solid line), the signal peaks for energy per water molecule close to 3 eV.^4^ We performed four simulations with the new potential to shed light on this phenomenon. The impact conditions had been chosen to be consistent with experimental values. We had chosen three (H_2_O)*_n_* projectiles with energy per water molecule equal to 5 eV (*n* = 4000), 2.85 eV (*n* = 7000), and 2 eV (*n* = 10000), which impacted, at an incidence angle of 45°, a hemispherical trehalose sample with a diameter of 60 nm. Additionally, we also modeled the impact of an even larger cluster, (H_2_O)_25000_ (*E*/*n* = 0.8 eV). The initial temperature of the sample was 0 K. Each simulation was performed for 50 ps, which was sufficient for sputtering processes to conclude. Other simulations settings can be found in the Supporting Information.

Figure 3 shows snapshots from trajectories of all four impacts. The largest water cluster dissolves part of the sample while maintaining its cohesion (a). In the case of (H_2_O)_10000_ and (H_2_O)_7000_ (b and c, respectively), some of the trehalose molecules are emitted with a partial shell of water molecules. For the highest energy per molecule, the projectile disintegrates into a cloud of water molecules on impact. Individual trehalose molecules and chunks of the trehalose sample are ejected (d), as seen previously for argon gas cluster projectiles.^18^ Entire animations can be found in the Supporting Information.

**Figure 3:**
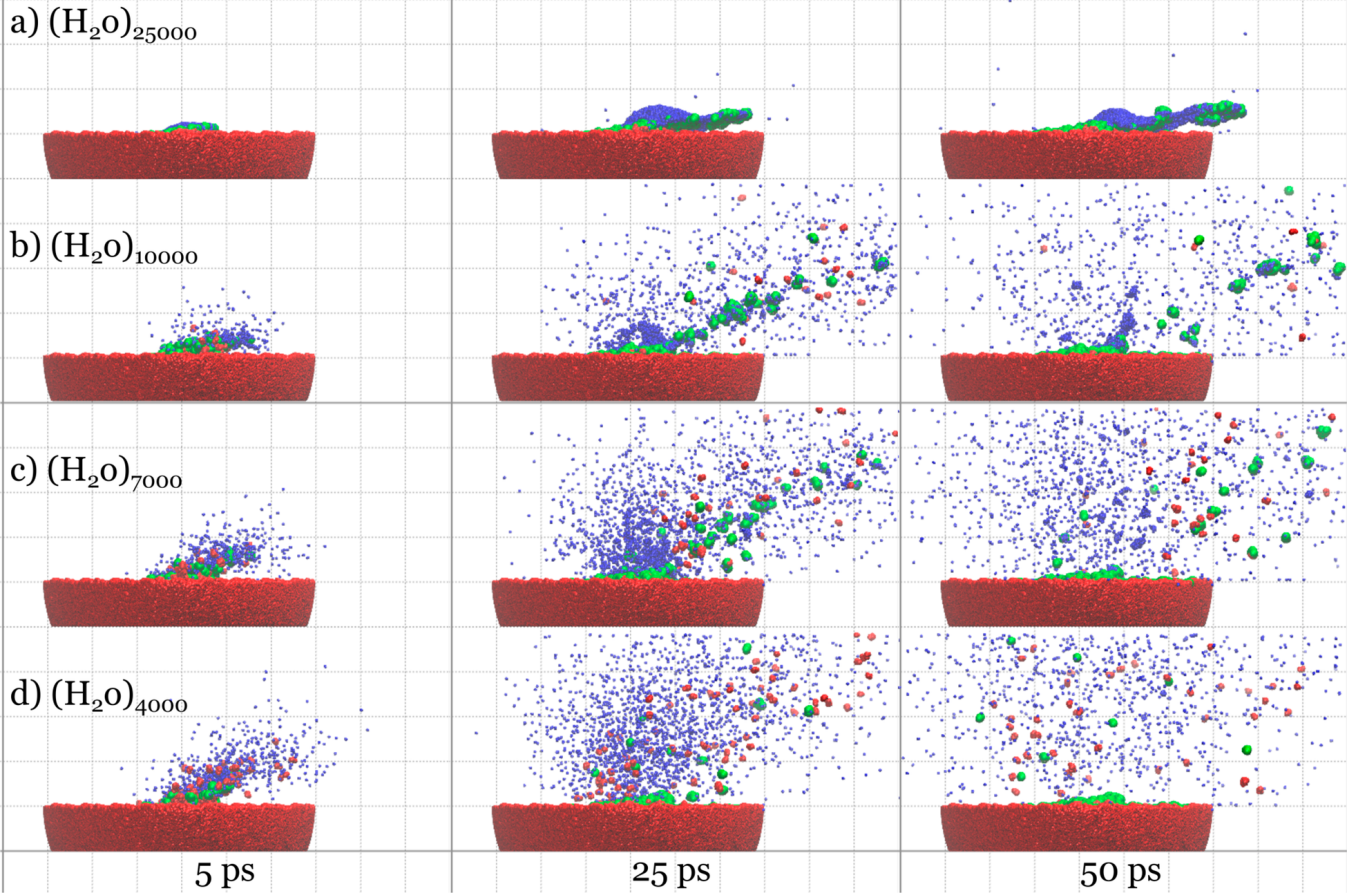
Side views at 5, 25, and 50 ps after the impact of 20 keV (a) (H_2_O)_25000_, (b) (H_2_O)_10000_, (c) (H_2_O)_7000_, and (d) (H_2_O)_4000_ projectiles at the trehalose sample. Trehalose molecules are colored red. Water is colored blue. Trehalose–water complexes are colored green. For the sake of clarity, the lower parts of the systems are not shown, and trehalose molecules are enlarged.

There is no correlation between the dependence of the amount of sputtered trehalose molecules (sputtering yield) on kinetic energy per projectile molecule (blue dashed line in Figure 4) and the experimental signal intensity (black solid line). However, when we included only sputtered trehalose–water complexes (defined as a trehalose ejected from the sample with at least one water molecule within 0.2 nm of it) in the sputtering yield (orange dotted line), the experimental and modeled dependences were remarkably similar. The only significant difference was the position of the maximum, which was shifted. There are several factors that can be responsible for this shift. For example, it has been reported that the surface roughness and sample temperature may influence sputtering phenomena.^19,20^ We will investigate this topic in the future.

**Figure 4:**
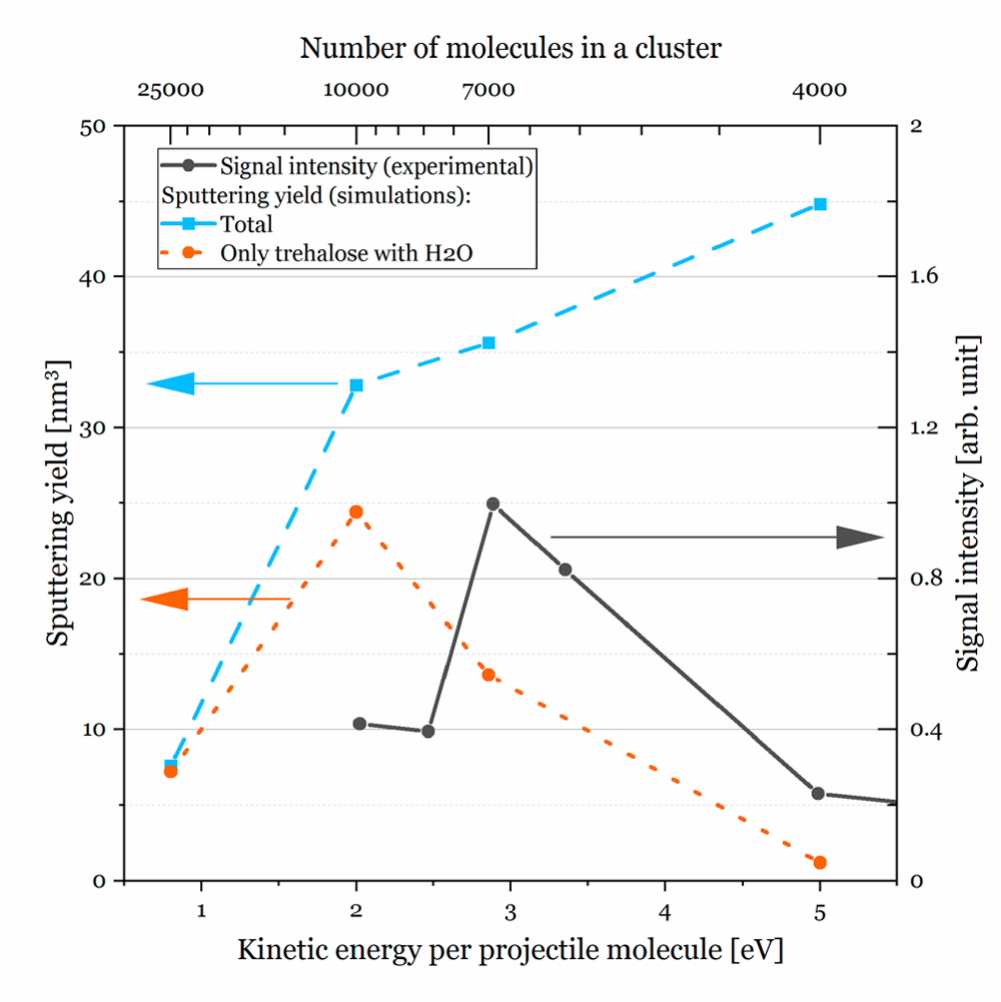
Signal enhancement for trehalose with an attached proton [M + H]^+^ bombarded by (H_2_O)_*n*_ clusters with a total kinetic energy of 20 keV (black solid line). Data were taken from ref (4). Sputtering yield for the same system predicted by ci-ReaxFF-CHO simulations: total (blue dashed line) and only emitted trehalose–water complexes (orange dotted line).

Although classical MD simulations cannot directly model ionization, our results point to a plausible explanation for the increase in the intensities of detected [M + H]^+^ ions between (H_2_O)_4000_ and (H_2_O)_10000_ projectiles. When the signal is enhanced, we observe an ejection of trehalose molecules with water around them, while trehalose is ejected separately in the case of (H_2_O)_4000_. The presence of vibrationally excited water molecules can facilitate a more efficient proton transfer,^21^ which may lead to an increase in the ionization probability. We cannot observe this phenomenon during our modeling because our potential (like every classical force field) cannot describe ionization. It is probable though that trehalose and nearby water molecules react with each other creating [M + H]^+^ ions. When the projectile size further increases (with a fixed total kinetic energy), trehalose molecules are not sputtered separately but are dissolved in the impacting cluster, creating a single very slow nanodroplet. The sputtering yield decreases significantly, which may explain the experimentally observed decrease in the detected [M + H]^+^ signal intensity.

We have created the first reactive potential without explicit electrostatic interactions that can model chemical reactions in C/H/O systems. ci-ReaxFF-CHO can accurately predict densities of a wide range of organic materials and water, surpassing ReaxFF-lg, a variant of ReaxFF with an additional term devoted to intermolecular interactions (Table 1). Additionally, ci-ReaxFF-CHO is more than twice as fast as standard parametrizations. It also achieves good accuracy in modeling chemical reactions. Finally, the incorporation of correct close-range repulsive barriers makes it a prime choice for modeling high-energy collisions present, for example, in sputtering simulations.

We used the new parametrization to gain insight into a mechanism of positive parent ion signal enhancement observed during the sputtering of the trehalose system by water cluster projectiles. We observed that the experimental increase in the intensity of the ion signal is correlated with emission of trehalose–water complexes. This topic will be further studied by modeling multiple bombardments and investigating the influence of temperature and surface roughness on the sputtering phenomena.

## Supporting Information Available

Potential file in a native ReaxFF format (TXT)
File with tabulated potential for short-range interactions in the LAMMPS format (TXT)
Example of usage of the charge-implicit ReaxFF with tabulated correction in LAMMPS (PDF)
Animation of trehalose bombardment by a (H_2_O)_*4000*_ water projectile (MP4)
Animation of trehalose bombardment by a (H_2_O)*_7000_* water projectile (MP4)
Animation of trehalose bombardment by a (H_2_O)*_10000_* water projectile (MP4)
Animation of trehalose bombardment by a (H_2_O)*_25000_* water projectile (MP4)
Details of the fitting procedure and simulation protocols used to validate the accuracy and the performance of the potential (PDF)

## References

[ref1] van Duin A. C. T.; Dasgupta S.; Lorant F.; Goddard W. A. ReaxFF: A Reactive Force Field for Hydrocarbons. J. Phys. Chem. A 2001, 105 (41), 9396–9409. 10.1021/jp004368u.

[ref2] Kański M.; Macia̧żek D.; Postawa Z.; Ashraf C. M.; van Duin A. C. T.; Garrison B. J. Development of a Charge-Implicit ReaxFF Potential for Hydrocarbon Systems. J. Phys. Chem. Lett. 2018, 9 (2), 359–363. 10.1021/acs.jpclett.7b03155. 29291618

[ref3] Stuart S. J.; Tutein A. B.; Harrison J. A. A reactive potential for hydrocarbons with intermolecular interactions. J. Chem. Phys. 2000, 112 (14), 6472–6486. 10.1063/1.481208.

[ref4] Sheraz née Rabbani S.; Berrueta Razo I.; Kohn T.; Lockyer N. P.; Vickerman J. C. Enhancing Ion Yields in Time-of-Flight-Secondary Ion Mass Spectrometry: A Comparative Study of Argon and Water Cluster Primary Beams. Anal. Chem. 2015, 87 (4), 2367–2374. 10.1021/ac504191m. 25588151

[ref5] Plimpton S. Fast Parallel Algorithms for Short-Range Molecular Dynamics. J. Comput. Phys. 1995, 117 (1), 1–19. 10.1006/jcph.1995.1039.

[ref6] Aktulga H. M.; Fogarty J. C.; Pandit S. A.; Grama A. Y. Parallel reactive molecular dynamics: Numerical methods and algorithmic techniques. Parallel Comput. 2012, 38 (4), 245–259. 10.1016/j.parco.2011.08.005.

[ref7] Humphrey W.; Dalke A.; Schulten K. VMD: Visual molecular dynamics. J. Mol. Graph. 1996, 14 (1), 33–38. 10.1016/0263-7855(96)00018-5. 8744570

[ref8] Chenoweth K.; van Duin A. C. T.; Goddard W. A. ReaxFF Reactive Force Field for Molecular Dynamics Simulations of Hydrocarbon Oxidation. J. Phys. Chem. A 2008, 112 (5), 1040–1053. 10.1021/jp709896w. 18197648

[ref9] Brandenburg J. G.; Maas T.; Grimme S. Benchmarking DFT and semiempirical methods on structures and lattice energies for ten ice polymorphs. J. Chem. Phys. 2015, 142 (12), 124104 10.1063/1.4916070. 25833562

[ref10] Hallbrucker A.; Mayer E. Calorimetric study of the vitrified liquid water to cubic ice phase transition. J. Phys. Chem. 1987, 91 (3), 503–505. 10.1021/j100287a002.

[ref11] Liu L.; Liu Y.; Zybin S. V.; Sun H.; Goddard W. A. ReaxFF-lg: Correction of the ReaxFF Reactive Force Field for London Dispersion, with Applications to the Equations of State for Energetic Materials. J. Phys. Chem. A 2011, 115 (40), 11016–11022. 10.1021/jp201599t. 21888351

[ref12] Lide D. R., Ed. CRC Handbook of Chemistry and Physics, 84th ed.; CRC Press: Boca Raton, FL, 2004.

[ref13] Zhang W.; van Duin A. C. T. Improvement of the ReaxFF Description for Functionalized Hydrocarbon/Water Weak Interactions in the Condensed Phase. J. Phys. Chem. B 2018, 122 (14), 4083–4092. 10.1021/acs.jpcb.8b01127. 29518340

[ref14] Ziegler J. F.; Biersack J. P. The Stopping and Range of Ions in Matter. In Treatise on Heavy-Ion Science: Astrophysics, Chemistry, and Condensed Matter; Bromley D. A., Ed.; Springer US, 1985; Vol. 6, pp 93–129.

[ref15] Zhang W.; van Duin A. C. T. Second-Generation ReaxFF Water Force Field: Improvements in the Description of Water Density and OH-Anion Diffusion. J. Phys. Chem. B 2017, 121 (24), 6021–6032. 10.1021/acs.jpcb.7b02548. 28570806

[ref16] Russo M. F.; Szakal C.; Kozole J.; Winograd N.; Garrison B. J. Sputtering Yields for C_60_ and Au_3_ Bombardment of Water Ice as a Function of Incident Kinetic Energy. Anal. Chem. 2007, 79 (12), 4493–4498. 10.1021/ac070105l. 17503768 PMC2553706

[ref17] Mahoney C. M. Cluster secondary ion mass spectrometry of polymers and related materials. Mass Spectrom. Rev. 2010, 29 (2), 247–293. 10.1002/mas.20233. 19449334

[ref18] Czerwinski B.; Rzeznik L.; Paruch R.; Garrison B. J.; Postawa Z. Effect of impact angle and projectile size on sputtering efficiency of solid benzene investigated by molecular dynamics simulations. Nucl. Instrum. Methods Phys. Res. B 2011, 269 (14), 1578–1581. 10.1016/j.nimb.2010.12.026.

[ref19] Tian H.; Macia̧żek D.; Postawa Z.; Garrison B. J.; Winograd N. C-O Bond Dissociation and Induced Chemical Ionization Using High Energy (CO2)_n_^+^ Gas Cluster Ion Beam. J. Am. Soc. Mass Spectrom. 2019, 30 (3), 476–481. 10.1007/s13361-018-2102-z. 30430438 PMC6417932

[ref20] Ben Hadj Mabrouk A.; Licitra C.; Chateauminois A.; Veillerot M. Effect of the molecular weight on the depth profiling of PMMA thin films using low□energy Cs^+^ sputtering. Surf. Interface Anal. 2021, 53 (10), 884–892. 10.1002/sia.6991.

[ref21] Leiderman P.; Gepshtein R.; Uritski A.; Genosar L.; Huppert D. Temperature Dependence of Excited-State Proton Transfer in Water Electrolyte Solutions and Water-Methanol Solutions. J. Phys. Chem. A 2006, 110 (29), 9039–9050. 10.1021/jp061226c. 16854014

