# Supplementary material for: Development of a Charge-Implicit ReaxFF for C/H/O Systems
Source: J Phys Chem Lett. 2022 Jan 12;13(2):628–33. doi: 10.1021/acs.jpclett.1c03867 (PMC8785188; doi:10.1021/acs.jpclett.1c03867)
Supplement: Supplementary file 3 — jz1c03867_si_003.pdf [file jz1c03867_si_003.pdf]

LAMMPS commands for using charge-implicit ReaxFF with tabularized correction potential

```
pair_style     hybrid/overlay reaxff NULL checkqeq no table linear 10000
pair_coeff     * * reaxff jz1c03867_si_001.txt C H O
pair_coeff     1 1 table jz1c03867_si_002.txt ireax_CHO_CC
pair_coeff     1 2 table jz1c03867_si_002.txt ireax_CHO_CH
pair_coeff     1 3 table jz1c03867_si_002.txt ireax_CHO_CO
pair_coeff     2 2 table jz1c03867_si_002.txt ireax_CHO_HH
pair_coeff     2 3 table jz1c03867_si_002.txt ireax_CHO_HO
pair_coeff     3 3 table jz1c03867_si_002.txt ireax_CHO_OO
```

There are 3 atom types in this example: 1 – carbon, 2 – hydrogen, 3 – oxygen. ReaxFF is splined with the correction potential tabularized in file “jz1c03867_si_002.txt” which is a part of the supporting information. Charge equilibration check is disabled (“checkqeq no”).
