# Supplementary material for: Development of a Charge-Implicit ReaxFF for C/H/O Systems
Source: J Phys Chem Lett. 2022 Jan 12;13(2):628–33. doi: 10.1021/acs.jpclett.1c03867 (PMC8785188; doi:10.1021/acs.jpclett.1c03867)
Supplement: Supplementary file 8 — jz1c03867_si_008.pdf [file jz1c03867_si_008.pdf]

Supporting Information

Development of a Charge-implicit ReaxFF for C/H/O Systems

Michał Kański[1], Sviatoslav Hrabar[1], Adri C.T. van Duin[2], Zbigniew Postawa[1,]*

[1] Smoluchowski Institute of Physics, Jagiellonian University, Łojasiewicza 11, 30-348 Kraków, Poland

[2] Pennsylvania State University, Department of Mechanical Engineering, University Park PA 16802, United States

[*] Email: zbigniew.postawa@uj.edu.pl

Density and heat of vaporization calculations

The heat of vaporization (H) can be calculated as [1]:

$$H = \frac{E_{gas} - E_{liquid}}{N} + RT,$$

where E_{gas} – the potential energy of gaseous sample, E_{liquid} – the potential energy of liquid sample, N – number of atoms in the system, R – the gas constant, T – temperature.

The simulations start from a semi-gaseous system which is compressed using a Nosé-Hoover barostat with target pressure equal to 1,000 atm. The temperature of the sample is maintained by a Nosé-Hoover thermostat. Molecules agglomerate during the compression step creating a condensed system. Subsequently, the pressure is gradually decreased to 1 atm and this pressure is maintained to achieve equilibration. The sample remains in the condensed state after

this stage. The density and potential energy of the system (E_{liquid}) is averaged during the next step. After that, the intermolecular interactions are disabled which effectively creates a gaseous system, whose potential energy (E_{gas}) is averaged after a sufficiently long equilibration.

The exact stages of the procedure are given below, along with the time of each step.

1. Create a primitive cubic molecular system with density 10 times lower than the experimental value.
2. Compress the system up to 1,000 atm (20 ps).
3. Maintain the pressure for 60 ps.
4. Relax system to 1 atm (20 ps).
5. Equilibrate the sample for 900 ps.
6. Average the density and potential energy of the system (E_{liquid}) for 100 ps.
7. Disable intermolecular interactions.
8. Perform an NVT simulation for 1 ps.
9. Average the potential energy of the system (E_{gas}) for 10 ps.

The number of molecules varied between systems and was chosen so that the predicted size of the equilibrated system in every dimension was at least four times higher than the cutoff of the potential. The damping constants of Nosé-Hoover thermostat and barostat were equal to 10 fs^{-1} and 100 fs^{-1}, respectively.

There were two exceptions from this procedure: the equilibration stage (5) for benzene was three times longer in order to achieve an equilibrium. Additionally, the simulation of freezing water started from the equilibrated water sample for 300 K (steps 1-4 were skipped), which was subsequently cooled to 77 K during a 200 ps simulation with Nosé-Hoover thermostat and barostat and 1 atm target pressure. Subsequently, the procedure was started from step 5.

Oxidation simulations

In order to test the new potential, an oxidation simulation was carried out for o-xylene. The modelling procedure was adapted from [2] and is given below:

1. An initial sample consisted of 100 O_2 molecules and a single o-xylene in a periodic cubic box with edge length equal to 2.5 nm.
2. The temperature of the sample was increased to 2500 K with the use of a Berendsen thermostat with the damping constant equal to 0.1 ps during a 10 ps simulation. After that, the system was equilibrated in 2500 K for 100 ps. The C-O and H-O interactions were disabled, so that no reactions would occur at this step.
3. Finally, the temperature was maintained at 2500 K by a Berendsen thermostat with a damping constant equal to 0.5 ps and the simulation was run till the oxidation concluded.

Sputtering simulations

Both water and trehalose samples used in sputtering simulations were hemispherical because the pressure waves induced by a cluster impact have spherical symmetry [3]. The size, density, and the number of atoms in the samples are given in Table S1. The rigid and stochastic layers with thicknesses of 0.7 and 2.5 nm, respectively, were used for preserving the shape of the system, and for suppressing reflections of pressure waves from the sample borders [3]. The simulations were run in 0 K target temperature for 50 ps, which was enough time for achieving saturation of the sputtering yield. Every simulation was performed once as the sputtering yield for cluster projectiles only weakly depends on the impact point [4].

Table S1 – Properties of the bombarded samples: potential used, diameter, number of atoms, and density. The numbers in parentheses are experimental densities for the systems.

Sample	Potential	Diameter [nm]	Number of atoms	Density [g/cm^3]
Frozen water	ci-ReaxFF-CHO	40	1,672,417	1.00 (0.94)
	ReaxFF-H_2O	40	1,743,400	1.05 (0.94)
Trehalose	ci-ReaxFF-CHO	60	6,346,437	1.42 (1.58)

Comparison of bond energies, valence barriers, and dihedral barriers between ci-ReaxFF-CHO and reference data

Figure S1 shows the comparison of ci-ReaxFF-CHO and ci-ReaxFF-CHO with tabularized correction to the ZBL potential (a, b). Without the tabulated potential, the ci-ReaxFF-CHO is in agreement with ZBL only for a narrow range of distances. This observation is especially true for the carbon-oxygen interaction. This cannot be avoided, since the ReaxFF formalism allows for specifying short-range repulsion parameters only for elements and uses automatic combination rules for pairs of different elements. Modifying this behavior would break compatibility with existing ReaxFF implementations. The addition of the tabulated potential increases the agreement to interatomic distances equal to about 0.01 nm regardless of the element. The transition from ci-ReaxFF-CHO to ci-ReaxFF-CHO with tabularized potential is smooth, thanks to the use of a splining region. An example of such region is shown in Figure S1c. The cutoffs for splining regions are given in Table S2.

Table S2 - Inner and outer cutoff values for splining region of tabulated correction potential

Pair of elements	Inner cutoff [nm]	Outer cutoff [nm]
CC	0.085	0.100
CH	0.070	0.090
CO	0.070	0.090
HH	0.035	0.050
HO	0.055	0.075
OO	0.060	0.085

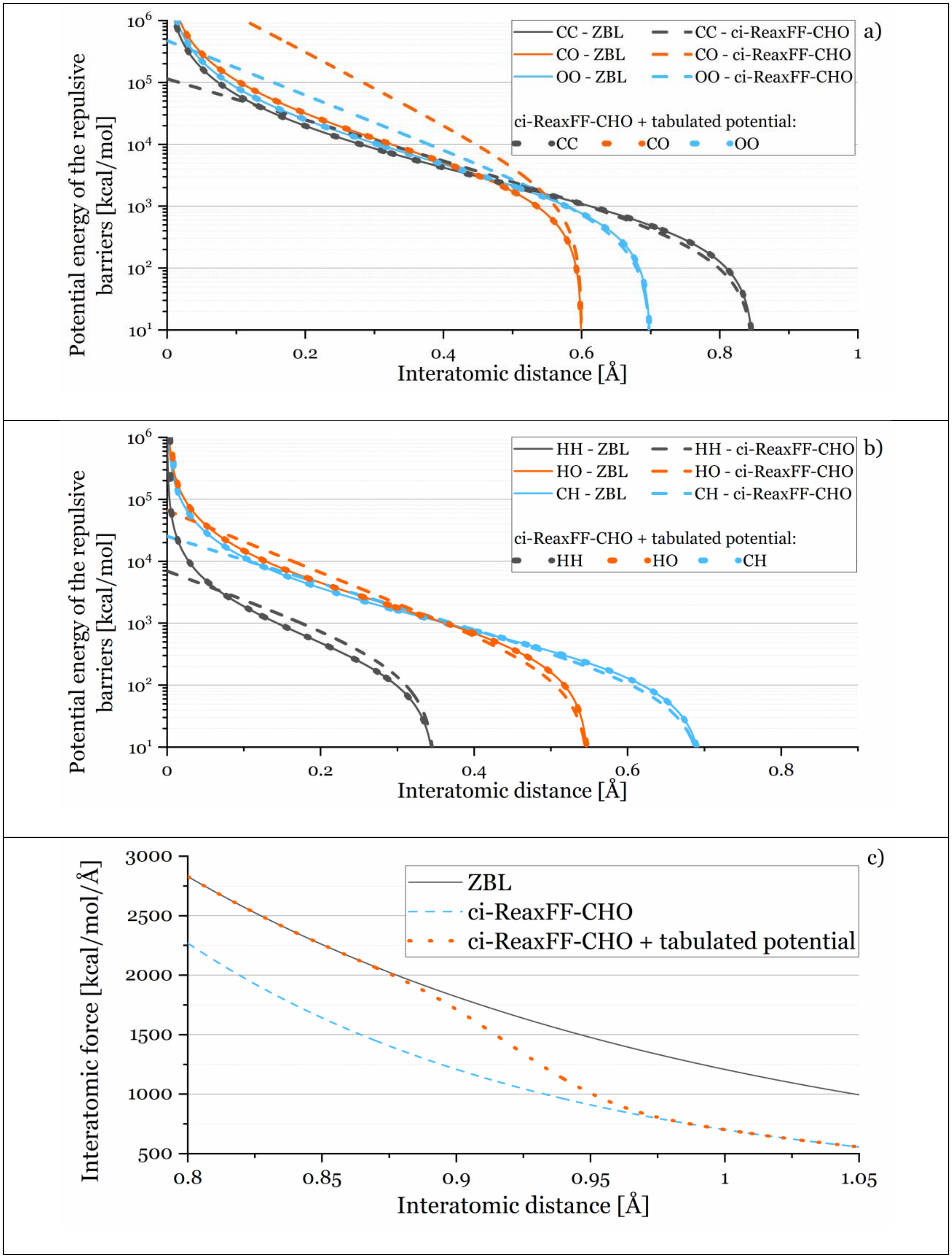

Figure S1 - Comparison of close-range repulsive barriers predicted by charge-implicit ReaxFF and ZBL potential (a,b) and the splining area for the C-C interaction (c).

Table S3 contains a comparison between DFT data (QM) taken from [2] and values predicted by ci-ReaxFF-CHO. An average deviation from reference data is 17%, compared to 16% for ReaxFF-2008 [2]. The bond dissociation curves used in the training process are in Figures S2-S4. Figures S5-S7 and Figures S8-S14 contain a comparison of valence and dihedral angle barriers, respectively. Other reactions curves are shown in Figures S15-S17. For QM data, the value in each graph is relative to a structure with minimum potential energy. The ci-ReaxFF-CHO values are relative to the potential energy, predicted by the potential of the same structure.

Table S3 - Comparison of reaction energies used in the training process.

C-H bond dissociation			ci-reaxFF-CHO	QM
$CH_x(OH)_y(CH_3)_z \rightarrow CH_{x-1}(OH)_y(CH_3)_z + H$, where x+y+z=4				
x	y	z		
1	0	3	91.4	100.7
1	1	2	94.9	95.9
1	2	1	109.5	96.8
1	3	0	91.4	100.0
2	0	2	93.5	99.1
2	1	1	95.3	98.8
2	2	0	91.1	103.1
3	0	1	99.4	100.2
3	1	0	97.0	107.0
4	0	0	107.9	111.6
C-OH bond dissociation			ci-reaxFF-CHO	QM
$CH_x(OH)_y(CH_3)_z \rightarrow CH_x(OH)_{y-1}(CH_3)_z + OH$, where x+y+z=4				
x	y	z		
0	1	3	88.7	92.7
0	2	2	86.4	94.5
0	3	1	95.2	100.5
0	4	0	75.9	106.3
1	1	2	87.8	93.9
1	2	1	85.1	95.9
1	3	0	77.7	104.0
2	1	1	92.8	94.3
2	2	0	85.6	95.9
3	1	0	97.5	93.6

Dehydrogenation	ci-reaxFF-CHO	QM
C_4H_{10}->C_4+5H_2	-133.7	-150.2
$CH_3CH_2CH_2OH$->CH_3CH_2CH=O+H_2	-26.1	-21.0
CH_3CH_2CH=O->CH_3CH=C=O + H_2	-36.1	-30.2
CH_3CH=C=O->CH_2=C=C=O + H_2	-69.0	-80.4
C-O bond dissociation	ci-reaxFF-CHO	QM
H_3COH->H_3C + OH	-97.3	-95.8
H_2C=O -> H_2C + O	-179.0	-172.1
CO -> C + O	-267.9	-256.93
C=O bond dissociation	ci-reaxFF-CHO	QM
$(CH_3)_2C$=O->$(CH_3)_2C$ + O	148.2	183.7
CH_3CH=O->CH_3CH + O	173.0	183.2
H_2C=O->H_2C + O	178.4	180.5
HOCH=O -> HOCH + O	175.9	183.1
HOC(CH_3)=O-> HOC(CH_3) + O	146.4	177.4
$(HO)_2C$=O->$(HO)_2C$ + O	99.8	157.9
C=C bond dissociation	ci-reaxFF-CHO	QM
H_2C=CH_2 -> 2 CH_2	163.6	171.1
HOCH=CH_2->HOCH+CH_2	163.7	156.1
HOCH=CHOH -> 2 CHOH	152.8	122.7
$(OH)_2C$=CHOH -> $(OH)_2C$ + CHOH	156.7	91.6
$(OH)_2C$=C$(OH)_2$-> 2C$(OH)_2$	145.4	52.6
O-O bond dissociation	ci-reaxFF-CHO	QM
HOOH -> HOO + H	75.3	89.0
HOO -> O_2 + H	56.9	53.6
H_3CO-OCH_3 -> H_3COO + CH_3	70.7	65.0
H_3CO-OCH_3 -> 2 OCH_3	26.4	35.7
HO-OCH_3 -> HO + OCH_3	40.2	43.9
HO-OCH_3 -> HOO + CH_3	76.2	66.9
H_3C-O-OH -> H_3C-O-O + H	75.2	85.7
OOO + O -> 2 O_2	104.4	107.8
C-H bond dissociation	ci-reaxFF-CHO	QM
H_2CCH=CH_2 -> H_2C-CH-CH + H	-83.5	-95.5
H_3COH -> H_2COH + H	-97.4	-96.06
H_2C=O -> HCO + H	-98.8	-88.04
HCOOH -> HOC=O + H	-92.0	-96.6
Other Reactions	ci-reaxFF-CHO	QM
H_3COH -> H_3CO + H	-88.5	-104
HCOOH -> HC-O-O + H	-65.5	-114.6
H_2C=O -> HC-OH	-79.0	-50
HCOOH -> C$(OH)_2$	-73.4	-43.4

Figure S2 - Bond energies for single (a), double (c), and triple (e) carbon-carbon bonds in ethane, ethene, and acetylene, respectively and carbon-hydrogen bond energies in ethane (b), ethene (d), and acetylene (f).

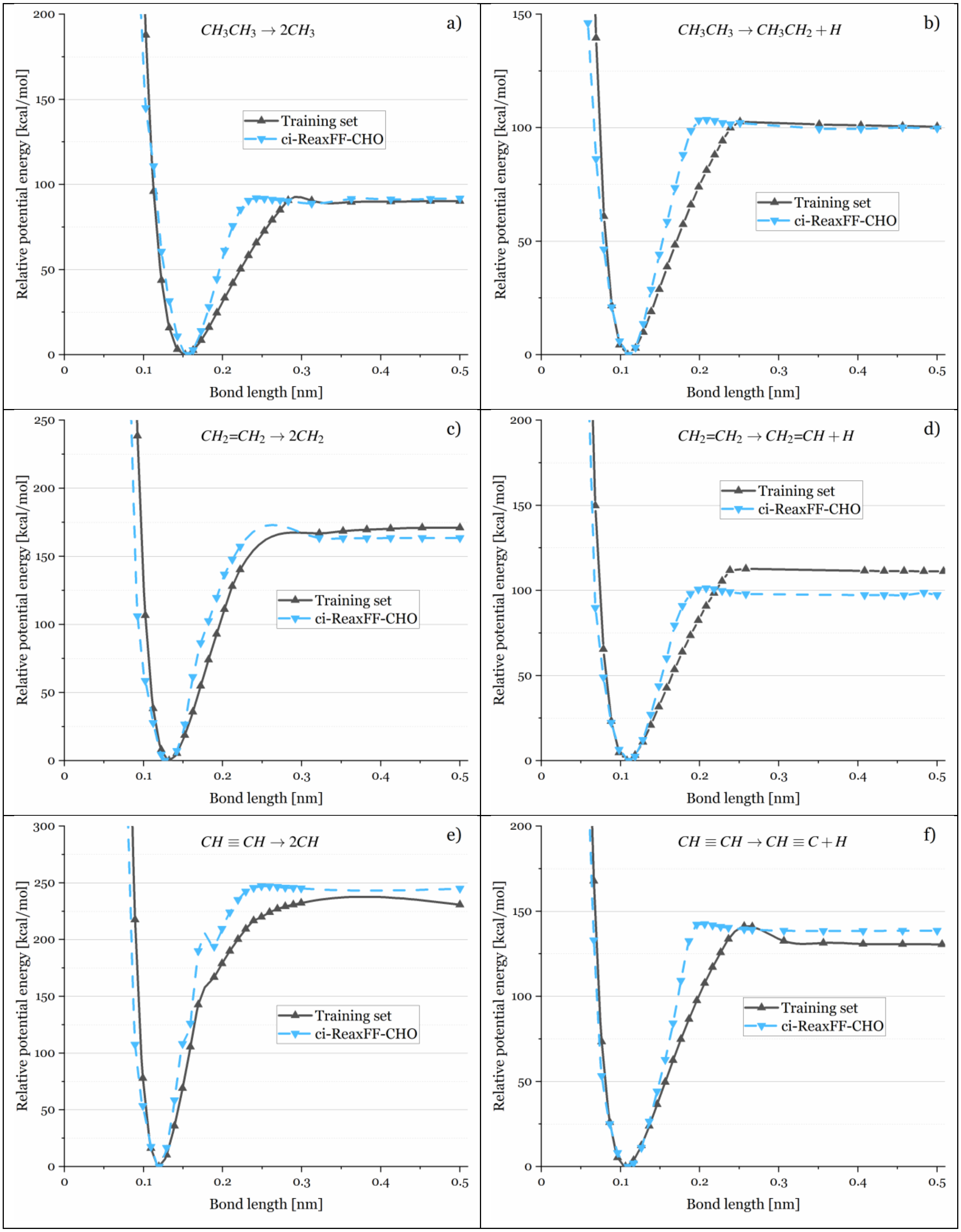

Figure S3 - Carbon-hydrogen bond energies in propane (a,b), single (c), double (d), and triple (e) carbon-oxygen bond energy in methanol, methanal, and carbon monoxide, respectively, and carbon-oxygen bond dissociation in CH₃CO

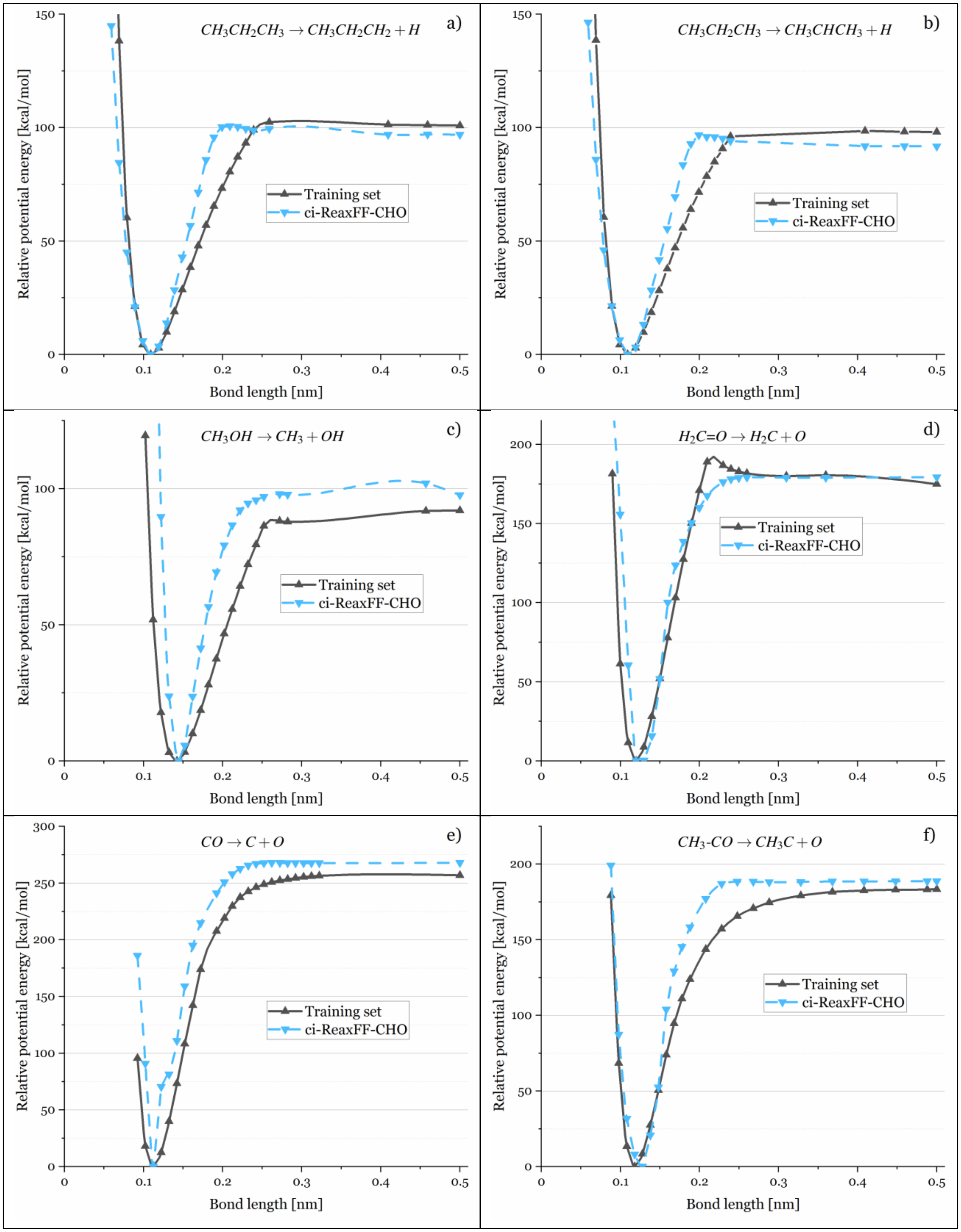

Figure S4 - Carbon-carbon bond dissociation curve in CH3CO (a), single (b) and double (c) oxygen-oxygen bond in molecular oxygen and hydrogen peroxide, respectively, oxygen-hydrogen bond dissociation in water (d), and H-H bond in hydrogene molecule..

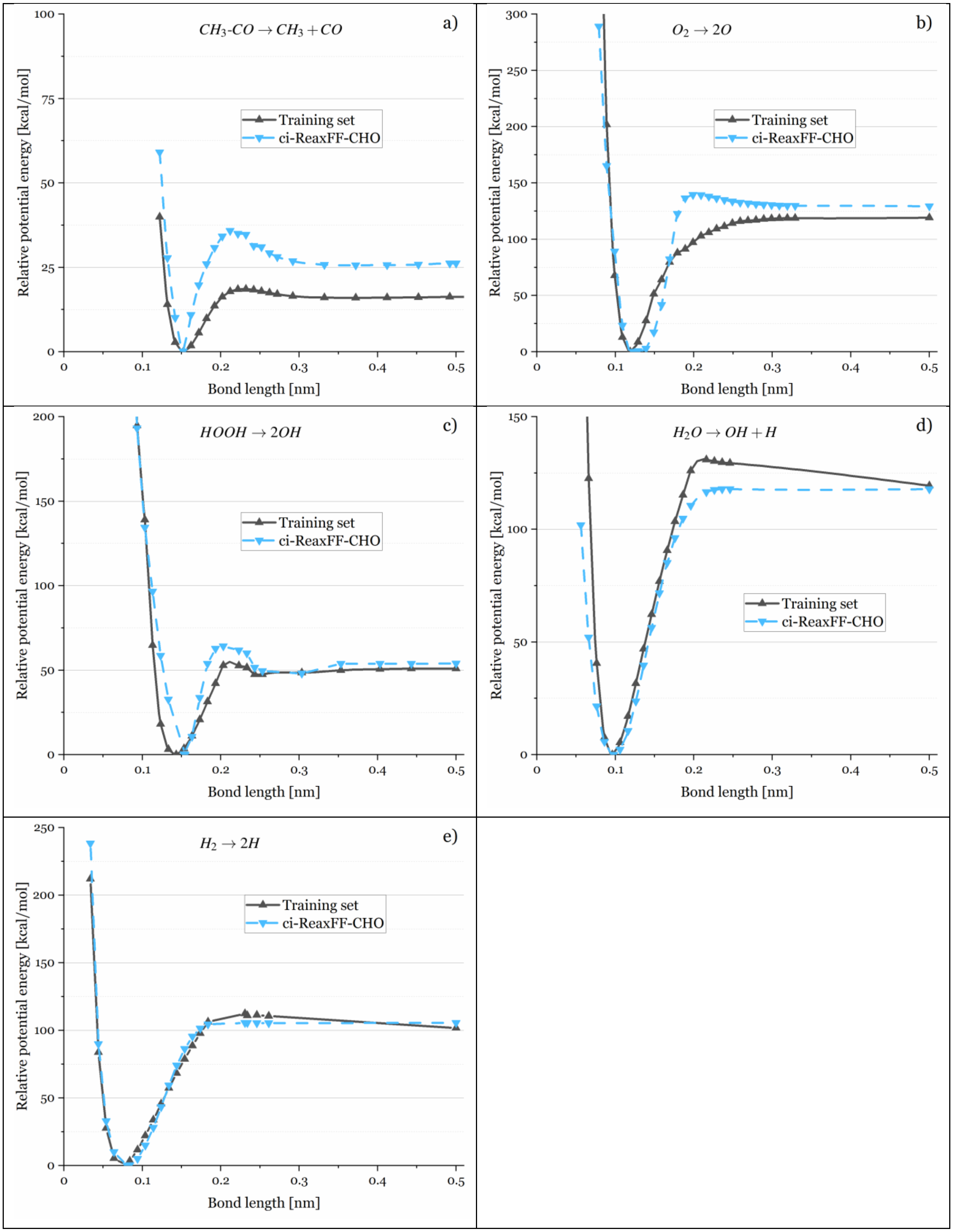

Figure S5 - C-C-C (a), H-C-H (b), C-C-H (c), C=C-H (d), C-C-O (e), C=C-O (f) valence angle barriers in propane, methane, ethane, ethene, ethanol, and ethenol, respectively.

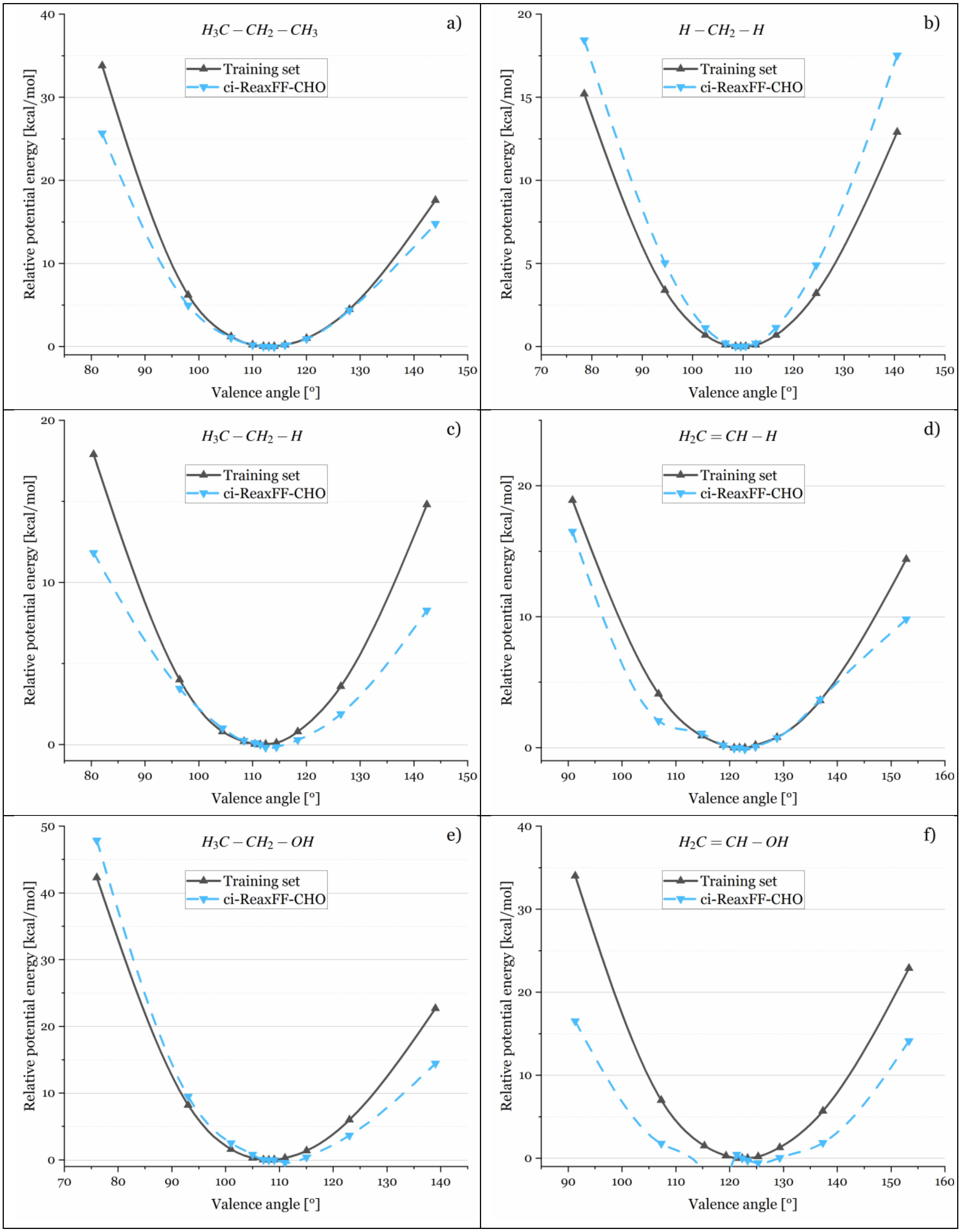

Figure S6 - C-O-C (a), C-O-H (b), C-O-O (c), H-O-H (d), H-C-O (e), O-C-O (f) valence angle barriers in dimethyl ether, methanol, methyl hydroperoxide, water, methanol, and methanediol, respectively.

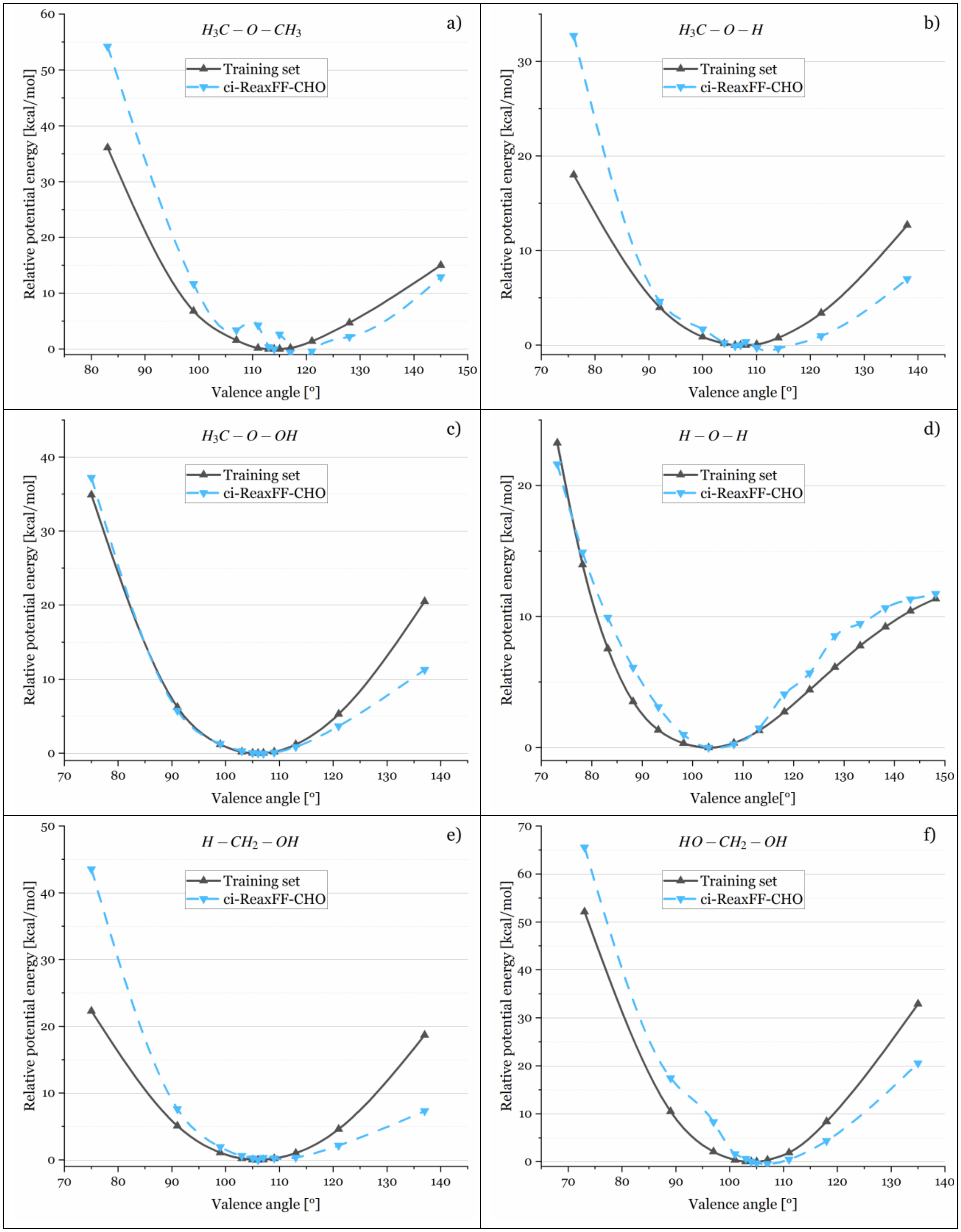

Figure S7 - O=C-C (a), O=C-H (b), O=C-O (c) valence angle barriers in ethanal, methanal, and formic acid, respectively.

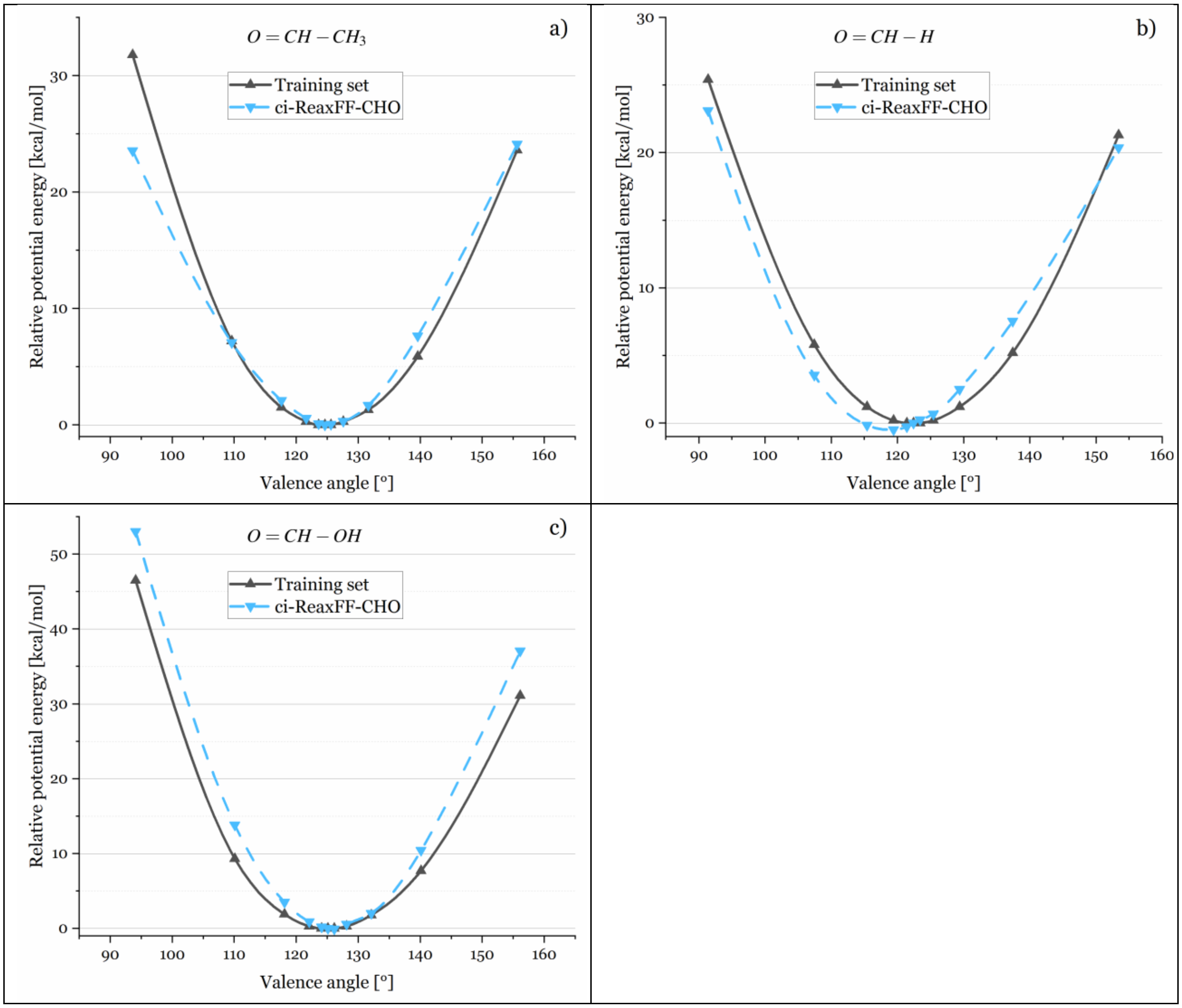

Figure S8 - C-C-C-C (a), C=C-C-C (b), C-C=C-C (c), C=C-C=C (d), C-C-C-H (e), C=C-C-H (f) dihedral angle barriers in butane, but-1-ene, but-2-ene, but-1,3-diene, propane, and propene, respectively.

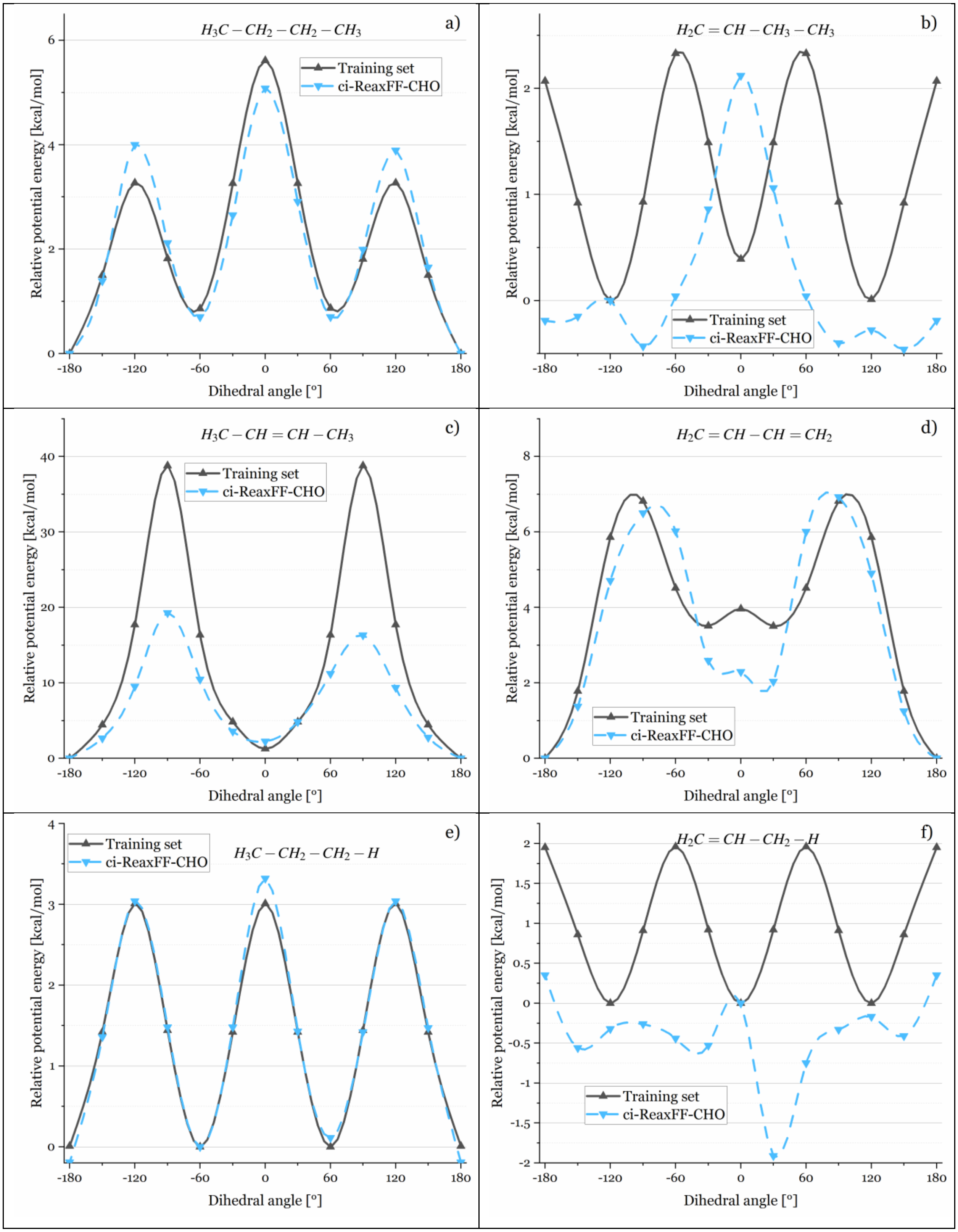

Figure S9 - C-C=C-H (a), C-C-C-O (b), C=C-C-O (c), C-C-C=O (d), C-C=C-O (e), C=C-C=O (f) dihedral angle barriers in propene, propanol, prop-2-enol, propanal, prop-1-enol, prop-2-enal, respectively.

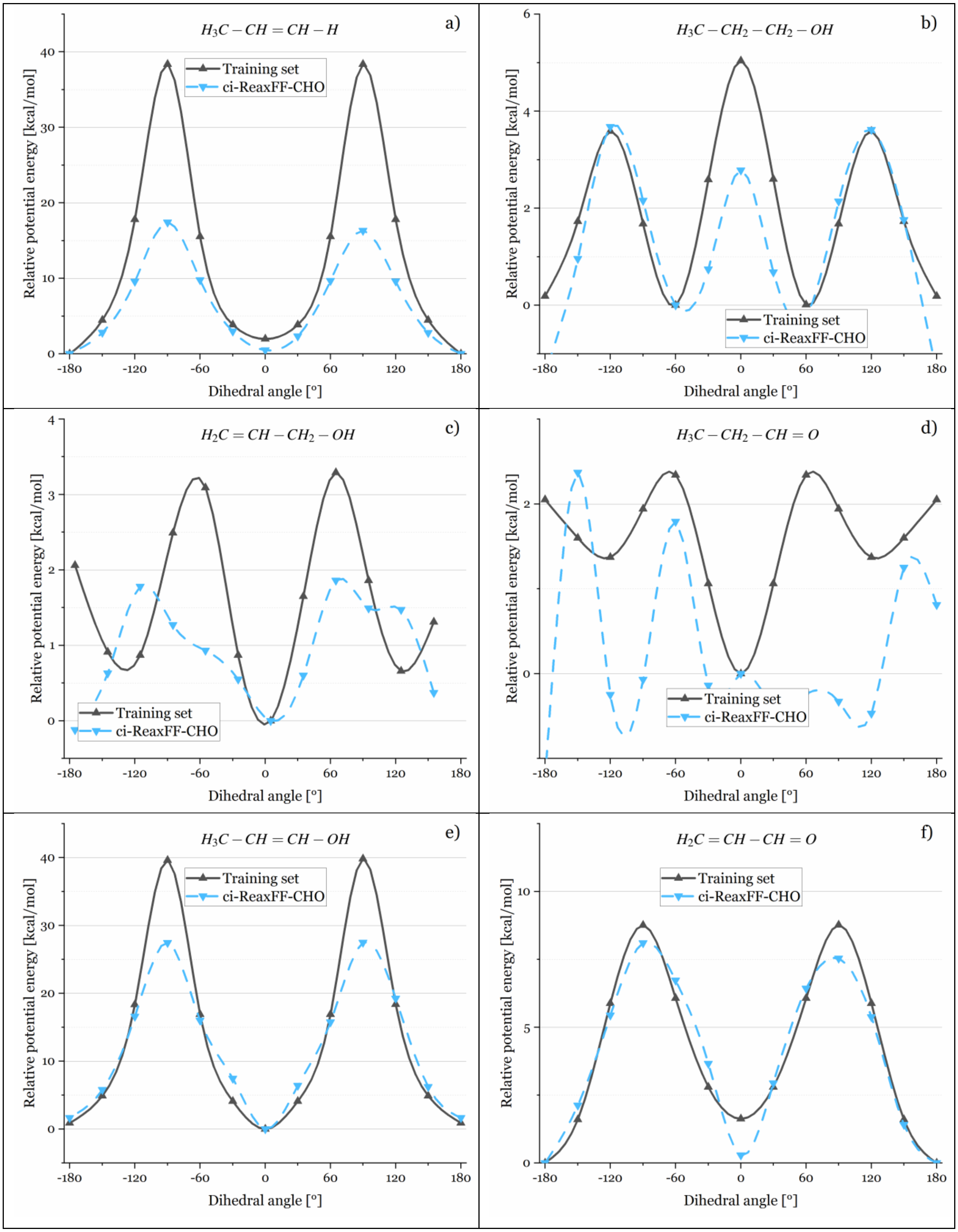

Figure S10 - C-C-O-C (a), C=C-O-C (b), C-C-O-H (c), C=C-O-H (d), C-O-C-O (e), C-O-C=O (f) dihedral angle barriers in ethyl methyl ether, methoxyethene, ethanol, ethenol, methoxymethanol, and methyl formate, respectively.

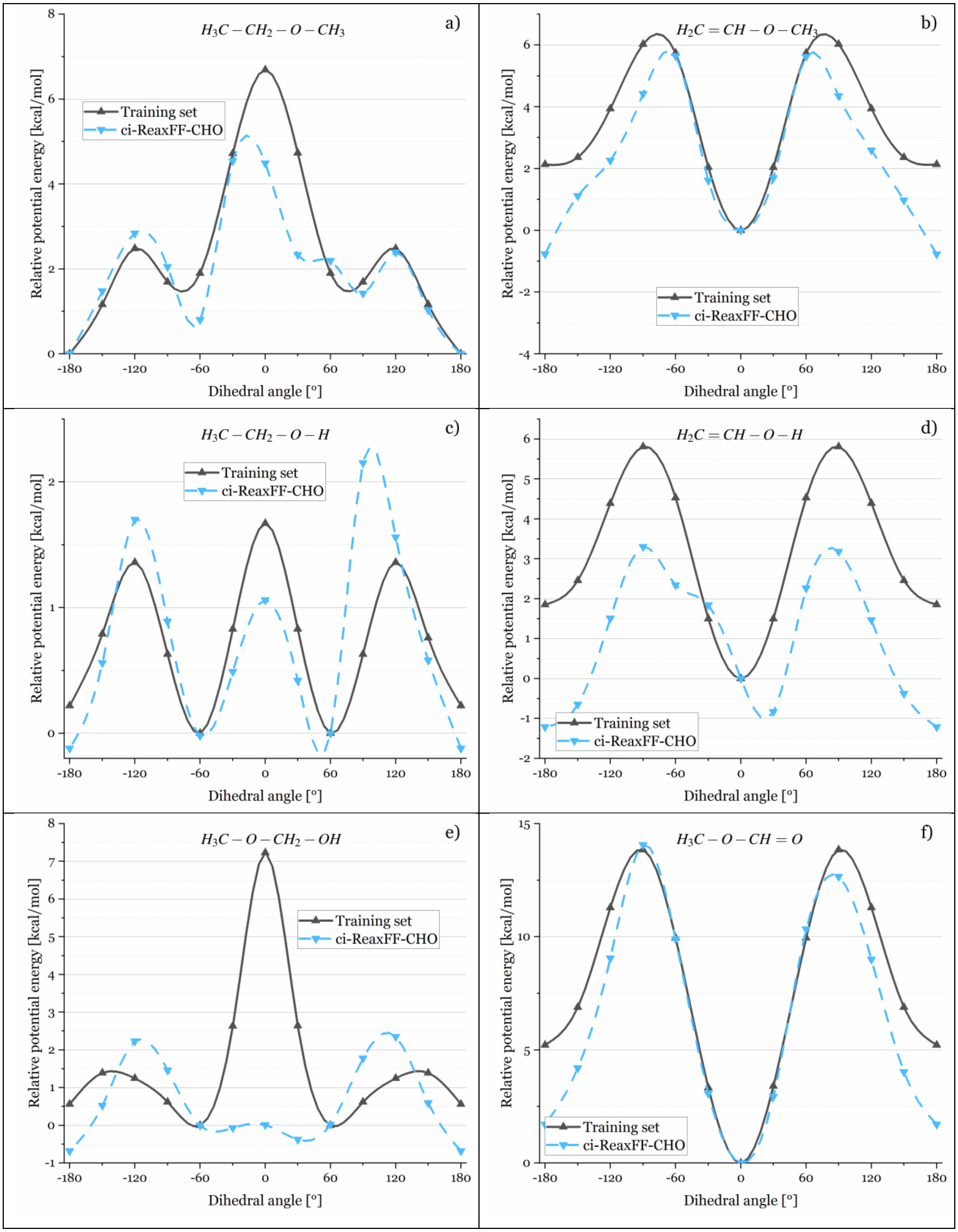

Figure S11 - C-O-C-H (a), C-O-O-C (b), C-O-O-O (c), O-O-O-O (d), H-C-C-H (e), H-C=C-H (f) dihedral angle barriers in dimethyl ether, dimethyl peroxide, methyl hydroperoxide, hydroxyperoxide, ethane, and ethene, respectively.

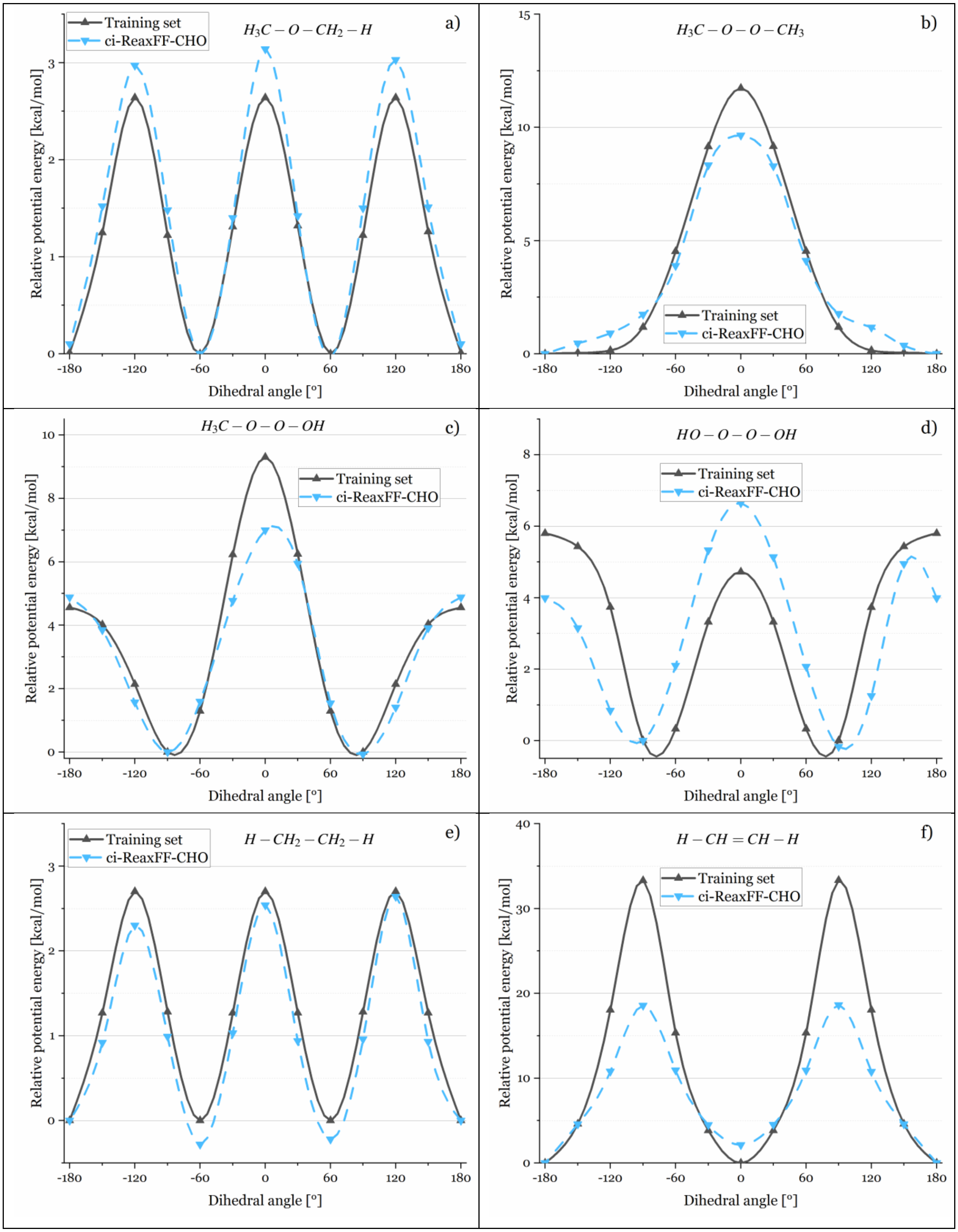

Figure S12 - H-C-C-O (a), H-C=C-O (b), H-C-O-H (c), H-C-O-O (d), H-O-C-O (e), H-O-C=O (f) dihedral angle barriers in ethanol, ethenol, methanol, methyl hydroperoxide, methanediol, and formic acid, respectively.

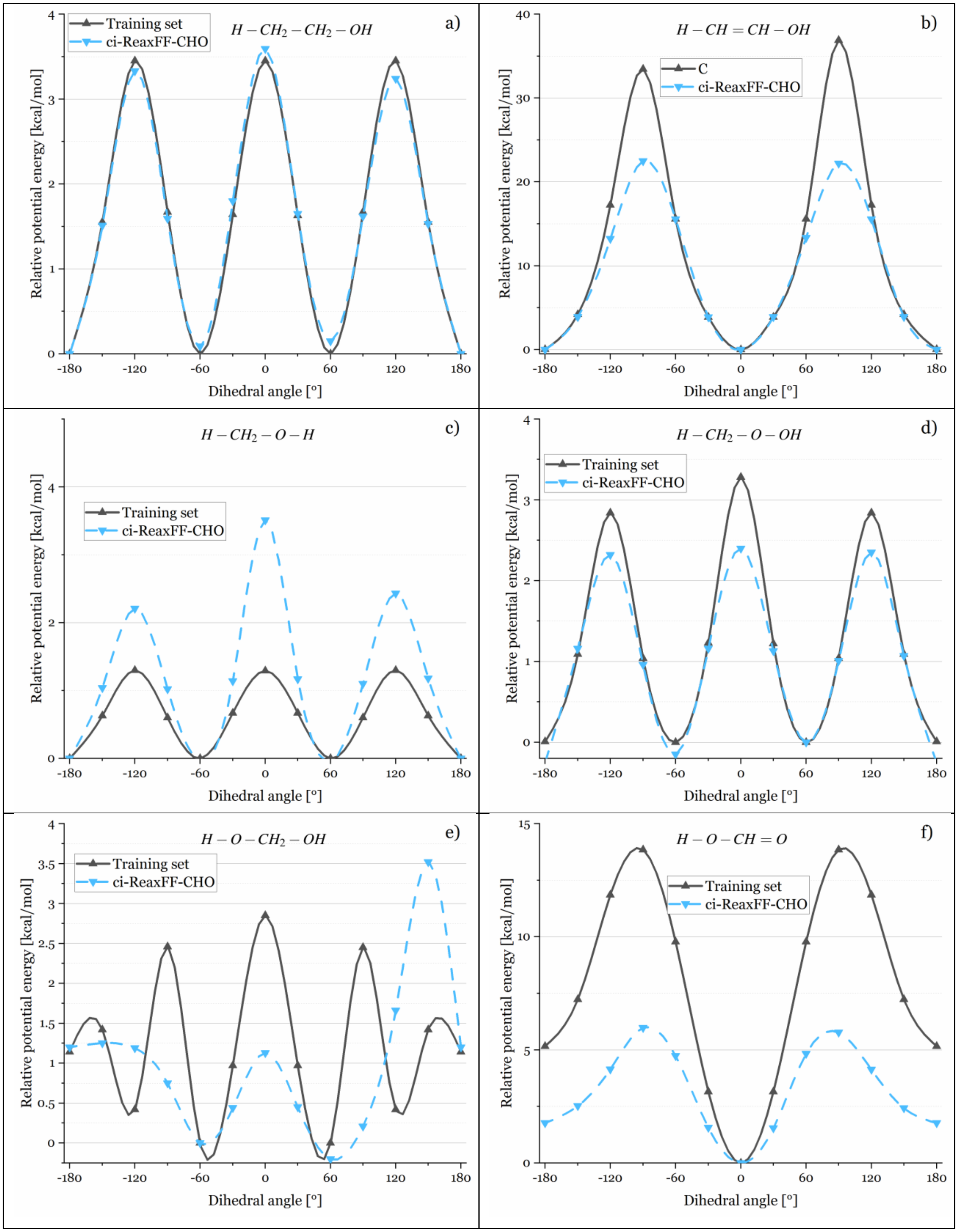

Figure S13 - H-O-O-H (a), H-O-O-O (b), O-C-C-O (c), O=C-O-O (d), O-C=C-O (e), O=C-C=O (f) dihedral angle barriers in hydrogen peroxide, trioxidane, ethane-1,2-diol, methaneperoxoic acid, ethene-1,2-diol, and oxaldehyde, respectively.

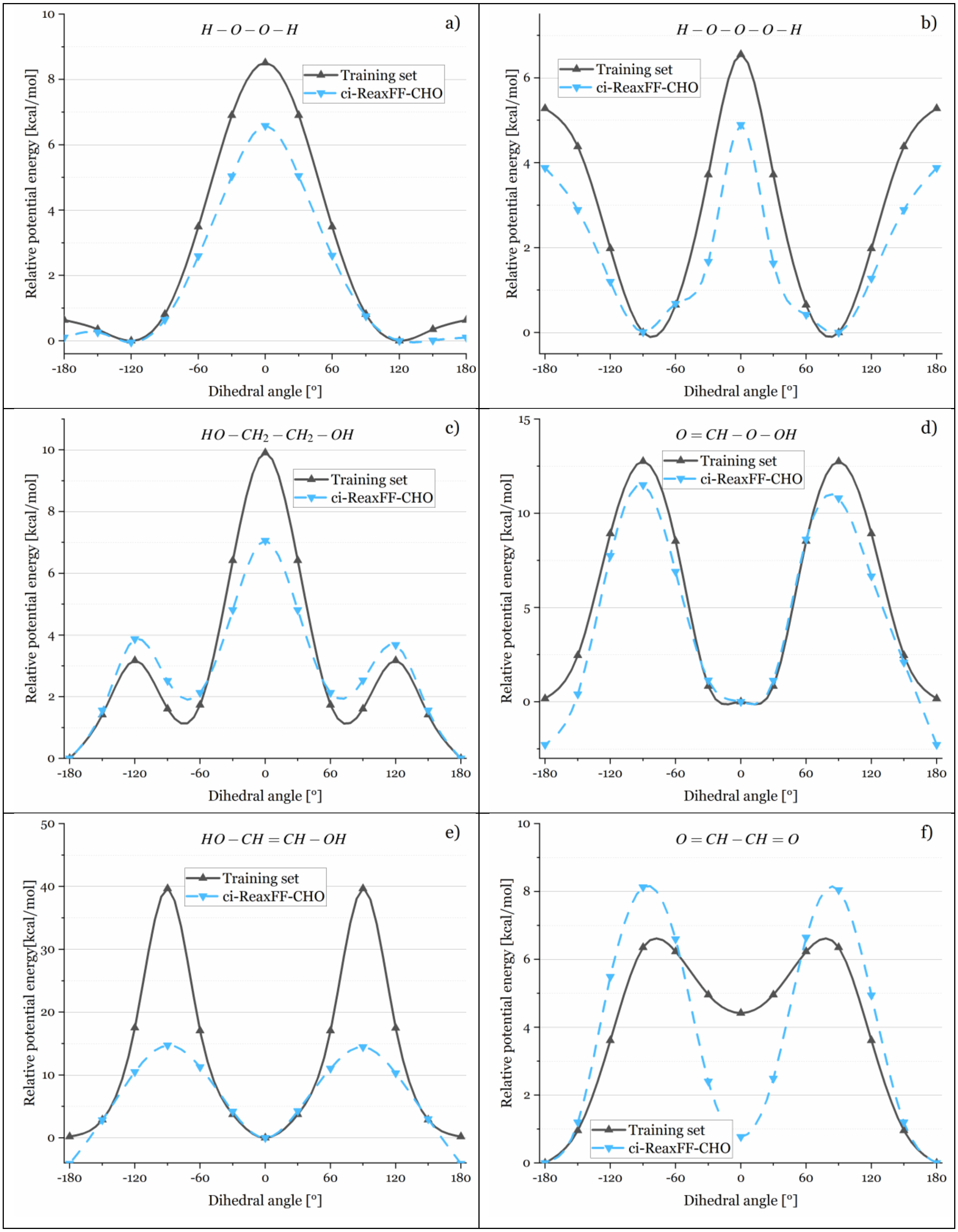

Figure S14 - O-C-O-O dihedral barrier in hydroperoxymethanol.

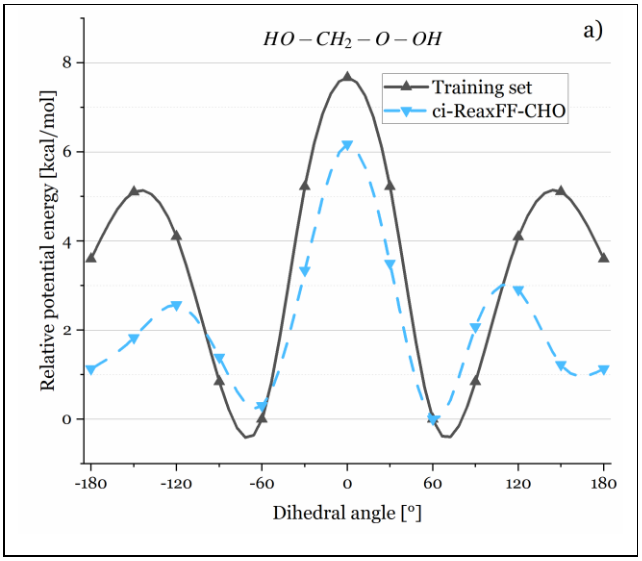

Figure S15 - Methylene group transfer between two methyl groups (a), hydrogen atom transfer between two methyl groups (b), hydrogen atom transfer between methyl group and hydrogen atom (c).

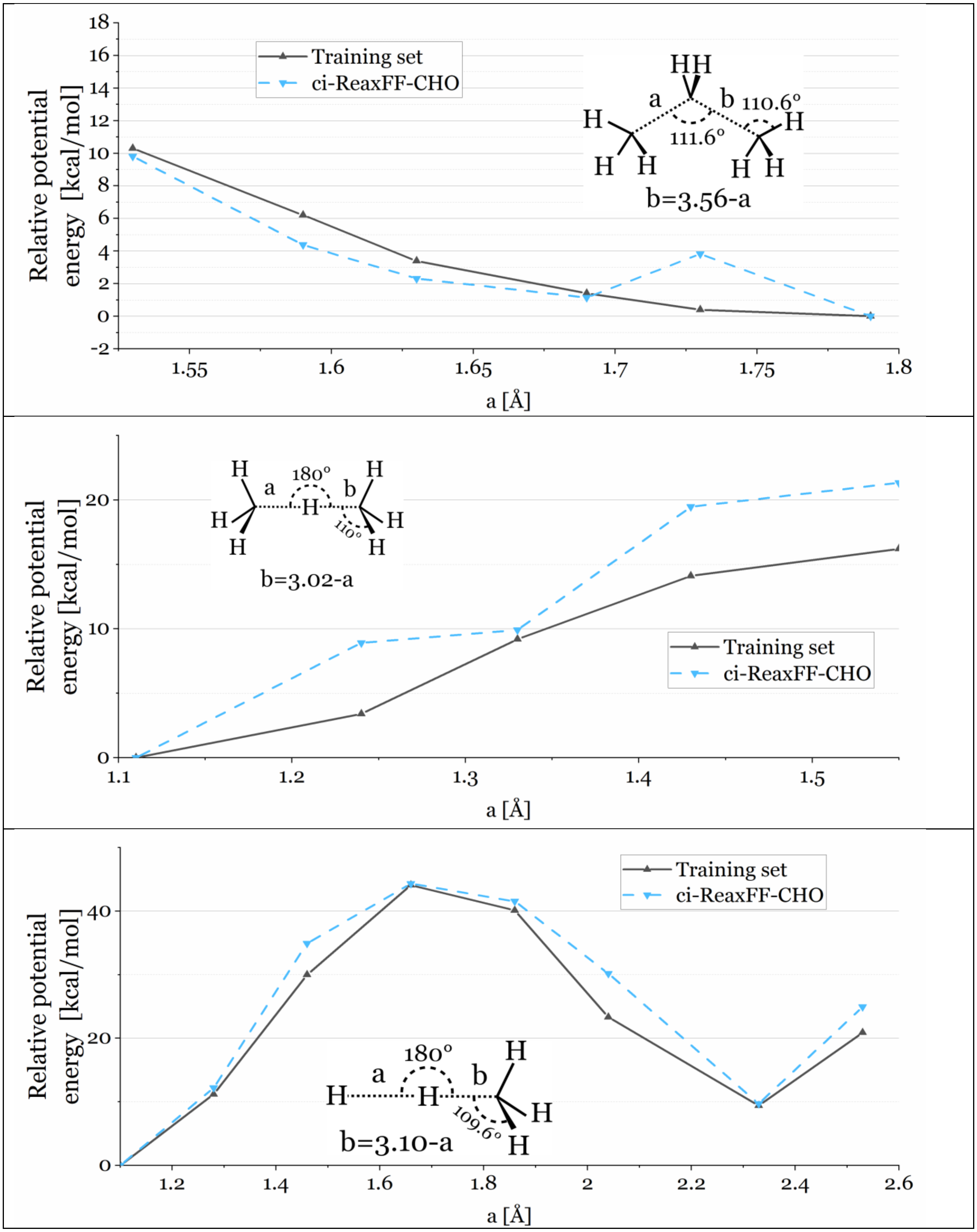

Figure S16 - C-C bond dissociation in n-butylbenzene radical (a, b) and in n-nonyl radical (c).

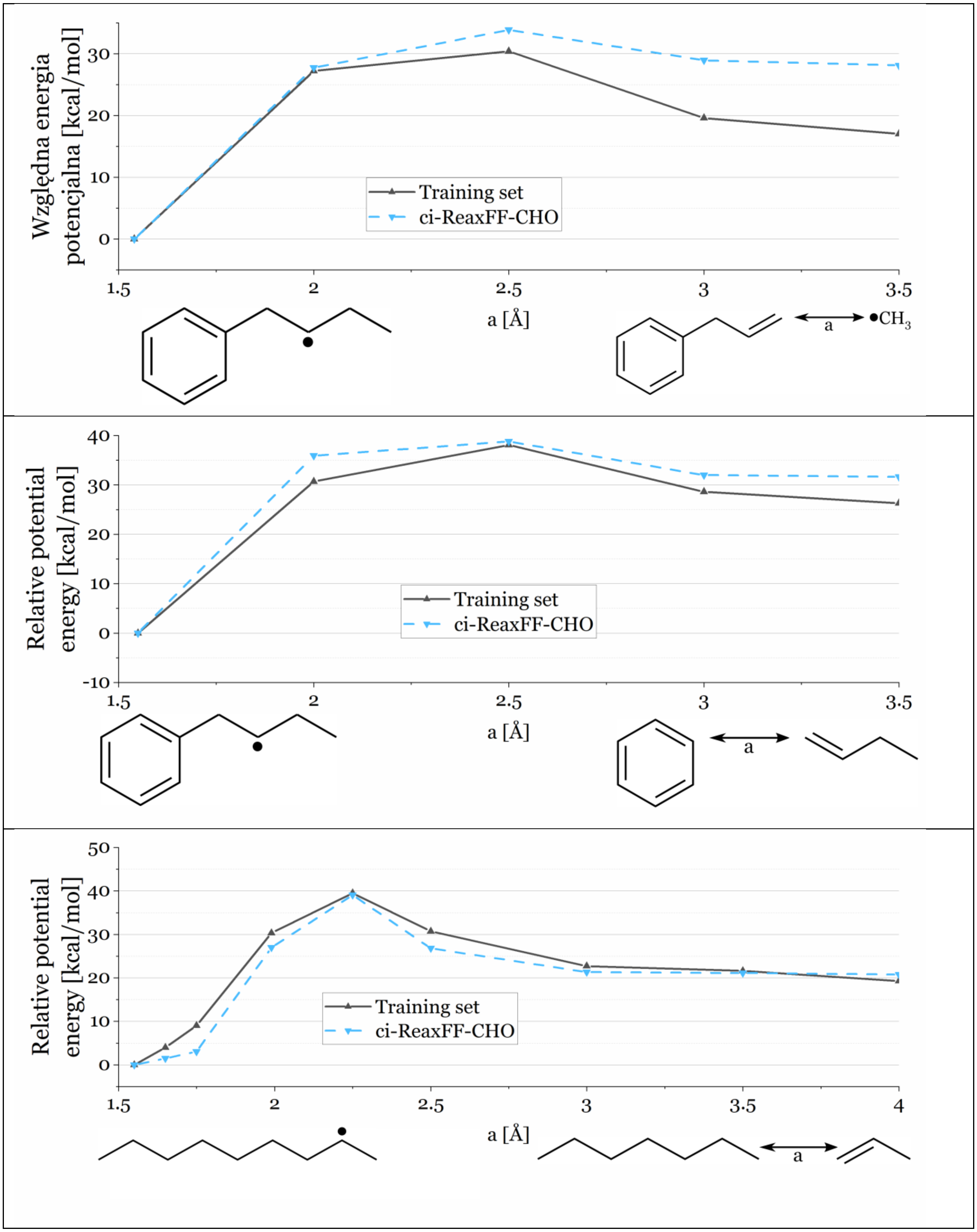

Figure S17 - Transition from cycloheaxatriene to benzene (a), Diels-Alder cycloaddition (b), energies of α, β, γ cleavages in butylbenzene (c).

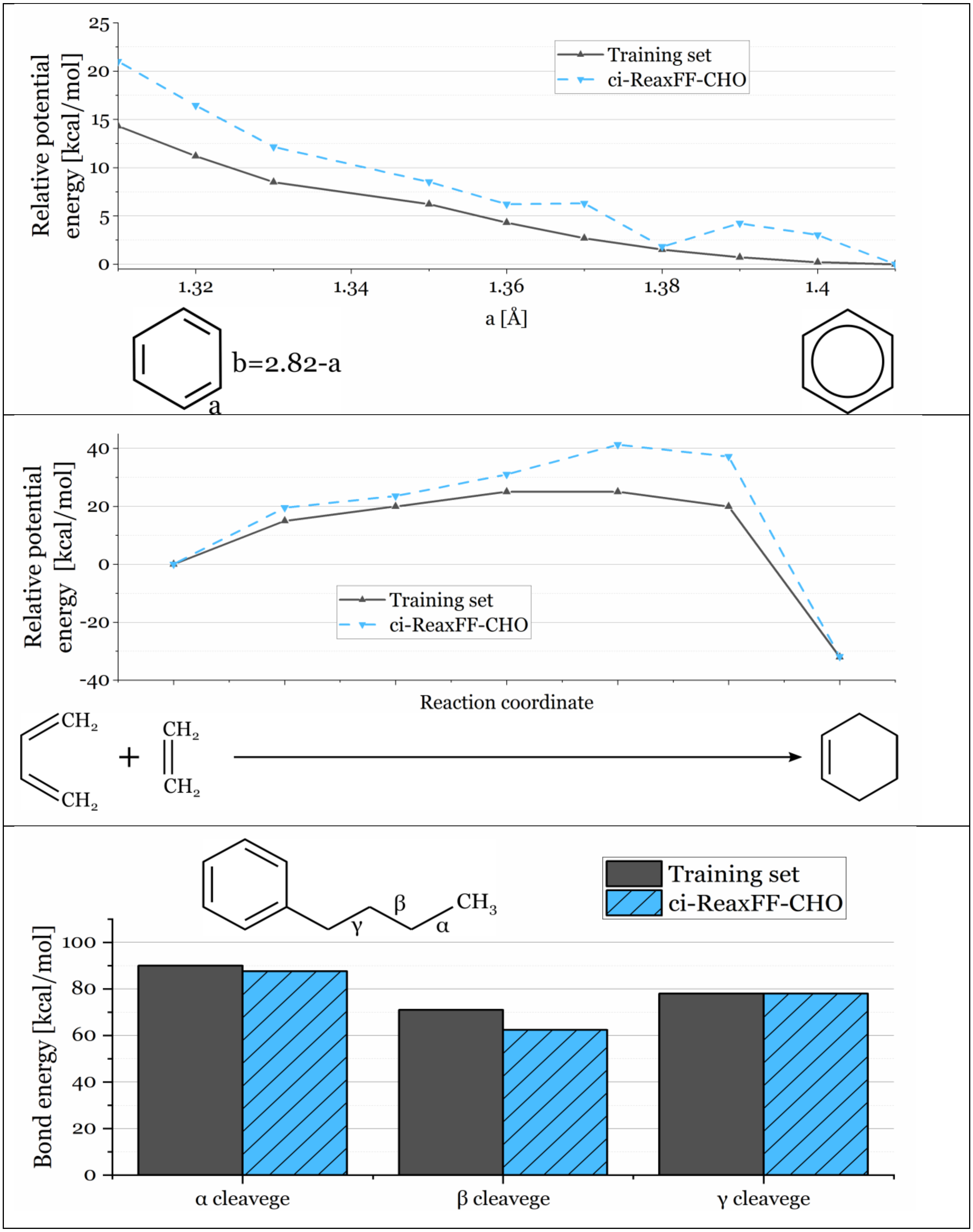

Simulations of trehalose bombarded by water clusters

Due to the size of the water clusters (Figure S18a), the diameter of the trehalose sample (Figure S18b) was increased to 60 nm.

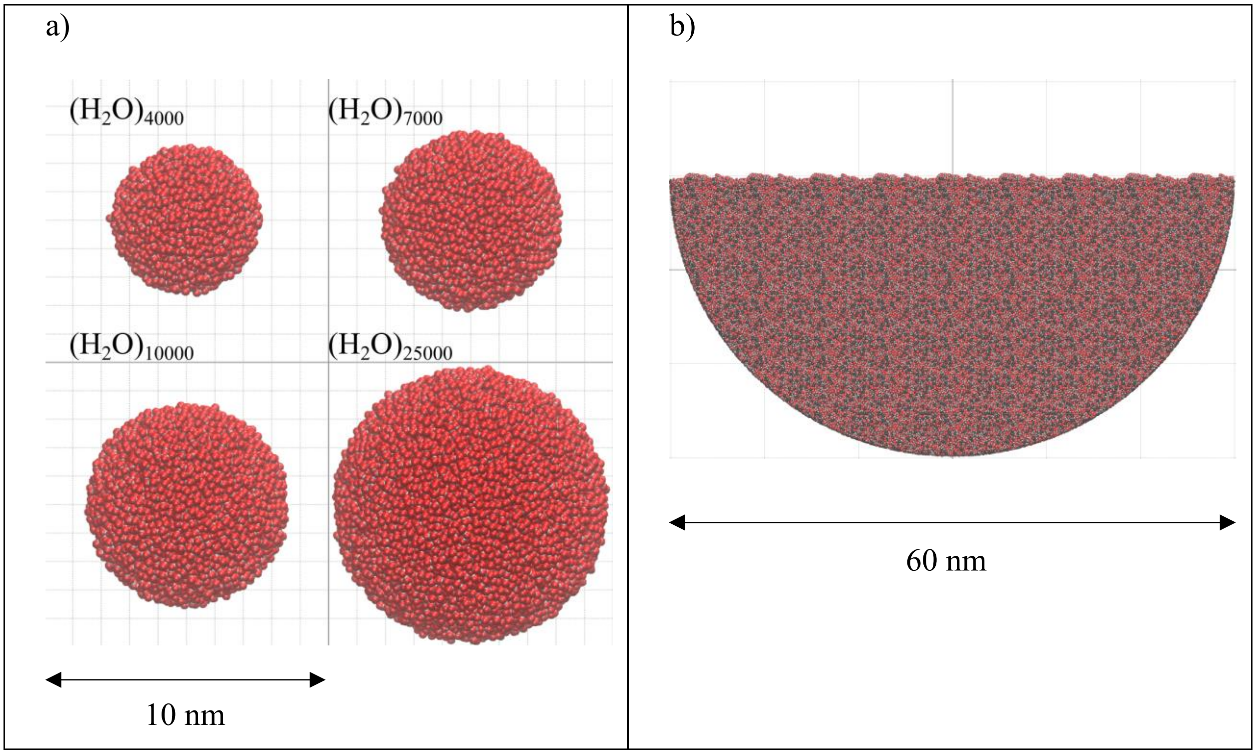

Figure S18 - $(H_2O)_n$ clusters (a) and a side view of the trehalose sample (b) used in sputtering simulations. Carbon atoms are black, hydrogen – white, and oxygen – red.

REFERENCES

(1) Kański, M.; Maciążek, D.; Postawa, Z.; Ashraf, C. M.; van Duin, A. C. T.; Garrison, B. J. Development of a Charge-Implicit ReaxFF Potential for Hydrocarbon Systems. *J. Phys. Chem. Lett.* **2018**, *9* (2), 359-363.

(2) Chenoweth, K.; van Duin, A. C. T.; Goddard, W. A. ReaxFF Reactive Force Field for Molecular Dynamics Simulations of Hydrocarbon Oxidation. *J. Phys. Chem. A* **2008**, *112* (5), 1040-1053.

(3) Czerwinski, B.; Rzeznik, L.; Paruch, R.; Garrison, B. J.; Postawa, Z. Effect of impact angle and projectile size on sputtering efficiency of solid benzene investigated by molecular dynamics simulations. *Nucl. Instrum. Methods Phys. Res. B* **2011**, *269* (14), 1578-1581.

(4) Czerwiński, B., Samson, R., Garrison, B.J., Winograd, N., Postawa, Z. Desorption of organic overlayers by Ga and C_{60} bombardment, *Vacuum* **2006**, *81*, 167-173.
